# A novel approach for human whole transcriptome analysis based on absolute gene expression of microarray data

**DOI:** 10.7717/peerj.4133

**Published:** 2017-12-08

**Authors:** Shirley Bikel, Leonor Jacobo-Albavera, Fausto Sánchez-Muñoz, Fernanda Cornejo-Granados, Samuel Canizales-Quinteros, Xavier Soberón, Rogerio R. Sotelo-Mundo, Blanca E. del Río-Navarro, Alfredo Mendoza-Vargas, Filiberto Sánchez, Adrian Ochoa-Leyva

**Affiliations:** 1 Departamento de Microbiología Molecular, Universidad Nacional Autónoma de México, Instituto de Biotecnología, Cuernavaca, Morelos, México; 2 Instituto Nacional de Medicina Genómica, Instituto Nacional de Medicina Genómica, México City, México; 3 Departamento de Inmunología, Instituto Nacional de Cardiología Ignacio Chávez (INCICh), México City, México; 4 Unidad de Genómica de Poblaciones Aplicada la Salud, Instituto Nacional de Medicina Genómica, México City, México; 5 Departamento de Ingeniería Celular y Biocatálisis, Instituto de Biotecnología, Universidad Nacional Autónoma de México, Cuernavaca, Morelos, México; 6 Laboratorio de Estructura Biomolecular, Centro de Investigación en Alimentación y Desarrollo, A.C. (CIAD), Hermosillo, Sonora, México; 7 Hospital Infantil de México Federico Gómez, Ciudad de México, Ciudad de México, México

**Keywords:** Microarray, Transcriptome, Human, Personalized medicine, Absolute gene expression, Transcriptomics, Leukocyte

## Abstract

**Background:**

In spite of the emergence of RNA sequencing (RNA-seq), microarrays remain in widespread use for gene expression analysis in the clinic. There are over 767,000 RNA microarrays from human samples in public repositories, which are an invaluable resource for biomedical research and personalized medicine. The absolute gene expression analysis allows the transcriptome profiling of all expressed genes under a specific biological condition without the need of a reference sample. However, the background fluorescence represents a challenge to determine the absolute gene expression in microarrays. Given that the Y chromosome is absent in female subjects, we used it as a new approach for absolute gene expression analysis in which the fluorescence of the Y chromosome genes of female subjects was used as the background fluorescence for all the probes in the microarray. This fluorescence was used to establish an absolute gene expression threshold, allowing the differentiation between expressed and non-expressed genes in microarrays.

**Methods:**

We extracted the RNA from 16 children leukocyte samples (nine males and seven females, ages 6–10 years). An Affymetrix Gene Chip Human Gene 1.0 ST Array was carried out for each sample and the fluorescence of 124 genes of the Y chromosome was used to calculate the absolute gene expression threshold. After that, several expressed and non-expressed genes according to our absolute gene expression threshold were compared against the expression obtained using real-time quantitative polymerase chain reaction (RT-qPCR).

**Results:**

From the 124 genes of the Y chromosome, three genes (DDX3Y, TXLNG2P and EIF1AY) that displayed significant differences between sexes were used to calculate the absolute gene expression threshold. Using this threshold, we selected 13 expressed and non-expressed genes and confirmed their expression level by RT-qPCR. Then, we selected the top 5% most expressed genes and found that several KEGG pathways were significantly enriched. Interestingly, these pathways were related to the typical functions of leukocytes cells, such as antigen processing and presentation and natural killer cell mediated cytotoxicity. We also applied this method to obtain the absolute gene expression threshold in already published microarray data of liver cells, where the top 5% expressed genes showed an enrichment of typical KEGG pathways for liver cells. Our results suggest that the three selected genes of the Y chromosome can be used to calculate an absolute gene expression threshold, allowing a transcriptome profiling of microarray data without the need of an additional reference experiment.

**Discussion:**

Our approach based on the establishment of a threshold for absolute gene expression analysis will allow a new way to analyze thousands of microarrays from public databases. This allows the study of different human diseases without the need of having additional samples for relative expression experiments.

## Introduction

The microarray is a powerful and cost-effective technology for the simultaneous detection of multiple mRNAs in a single reaction, obtaining the global gene expression of a whole cell or tissue  (Shyamsundar et al., 2005). This technology consists in a collection of DNA oligonucleotide spots, known as probes, attached to a solid surface where all genes of interest are represented with only one or several oligonucleotide probes. Then, after nucleotide hybridization by complementary base pairs between the probe and the target cDNA sequence the fluorescence intensity generated is interpreted as an indicator of the gene expression level and it is used to identify up-regulated and down-regulated genes (Lipshutz et al., 1999; Tang et al., 2007).

The microarrays are typically used to obtain the relative gene expression, i.e., which genes are differentially expressed in one condition compared to other. This implies the need of two microarrays to perform the expression analysis. However, the identification of absolute gene expression is not possible using a relative expression analysis. The identification of which genes are expressed in different tissues and cells will increase our understanding about the gene expression role in the human cell biology and disease (Pontén et al., 2009; Björling et al., 2008).

There are over 767,000 microarrays of RNA from human samples in public repositories such as the Gene Expression Omnibus (GEO), which are an invaluable resource for biomedical research and personalized medicine. For example, gene expression patterns, based on relative gene expression analysis have been used to make clinical predictions in several types of cancer. However, the majority of these microarray data have been used for relative expression analysis and minimal attention has been devoted to obtain an absolute measure of gene expression (McCall et al., 2013). The complexity in obtaining the absolute gene expression from microarrays is the different hybridization efficiency of each probe, which impacts in the fluorescence intensities. This is known as the “probe effect” and it introduces a bias in the fluorescence intensity with respect to the real expression level (Zilliox & Irizarry, 2007). This probe effect is typically canceled out by relative measures of gene expression (Irizarry et al., 2005). Several algorithms have been developed to overcome the probe effect measuring the absolute gene expression from microarray data (McCall et al., 2013; Seita et al., 2012). Here, we propose a novel approach to obtain the absolute gene expression from human microarray data based on the expression analysis of Y chromosome genes. This analysis allowed to establish a fluorescence threshold to distinguish between non-expressed (background fluorescence) and expressed genes (real fluorescence). We used this method to determine expressed and non-expressed genes in the transcriptomes of 16 human leukocyte samples and confirmed their expression level by RT-qPCR.

## Materials & Methods

### Ethics statement

Blood tissues were obtained from volunteers recruited from Hospital Infantil de México Federico Gomez. Parents or guardians of each child signed the consent form for participation in the project. The study was approved by the Ethics Committee of Hospital Infantil de México Federico Gomez (4000/536/2012).

### RNA extraction, microarray hybridization and data analysis

Total RNA from leukocytes of sixteen children (nine males and seven females, ages 6–10 years) was isolated using TRIzol (Invitrogen, Carlsbad, CA, USA). To avoid DNA carryover, samples were treated with recombinant DNAse I (Roche, Basel, Switzerland). The initial concentration of RNA was 1 µg for each sample and the quality was assessed using the Bioanalyzer 2100 (Agilent Technologies, Santa Clara, CA, USA). Polyadenylated controls were prepared and RNA was converted to complementary DNA (cDNA). *In vitro* transcription was performed for generating complementary RNA (cRNA). The cRNA was purified and quantified; cDNA was synthesized again and the remaining cRNA was hydrolyzed with RNase H. The cDNA was purified and quantified subsequently it was fragmented into 40–70 base pairs (bp) fragments. The cDNA fragments were labeled and hybridized to an Affymetrix Gene Chip Human Gene 1.0 ST Array for 16 h at 45 °C. This microarray contains probes for the analysis of about 28,869 genes, with an average of 26 probes of 25 oligonucleotides for each gene. After hybridization, microarrays were washed in the Fluidics Station (Affymetrix Gene Chip Fluidics Station 450 FS; Affymetrix, Santa Clara, CA, USA) following the manufacturer’s instructions and scanned using the System Affymetrix Gene Chip Scanner 3000 7G. This analysis was performed on the Affymetrix platform in the Genotyping and Expression Analysis Unit of the National Institute of Genomic Medicine (INMEGEN). Quality control of the microarray data was done using RMA Express (http://rmaexpress.bmbolstad.com/). The original array data was preprocessed with background adjustment, quartile normalization and PLM summarization method by robust multiarray average (RMA) using RMA Express (Irizarry et al., 2003). The raw expression data is available on the NCBI Gene Expression Omnibus (GEO) under accession number GSE89571.

### Selection of the Y chromosome genes for absolute gene expression threshold

The 124 genes of the Y chromosome were obtained using the Genome Browser bioinformatics tool (https://genome.ucsc.edu/). The fluorescence intensities of these genes were analyzed in a set of nine males and seven female microarrays (GSE89571) from Affymetrix Gene Chip Human Gene 1.0 ST Array, which were previously normalized by RMA (Irizarry et al., 2003). The non-parametric Mann Whitney test was performed to select the Y chromosome genes with fluorescence significantly different between the sexes. To calculate the absolute gene expression threshold, we used the *Z*-test to the *Z*-score at 95% confidence interval.

### RNA extraction and Real-time PCR

To perform the RT-qPCR expression analysis, 1 µg of total RNA was reverse-transcribed with TaqMan Reverse Transcription Reagents (Applied Biosystems, Foster City, CA, USA) using random hexamers according to the manufacturer’s protocol. One-tenth volume of each RT reaction was amplified with SYBR Green master mix (Roche, Basel, Switzerland) containing 0.5 mM of intron-spanning specific primers as well as Fast Start enzyme, PCR buffer and 3.5 mM MgCl_2_ (Lipshutz et al., 1999), in a final volume of 10 µl, the reactions were measured in a Light Cycler 2.0 real-time PCR detection system (Roche, Basel, Switzerland). Glyceraldehyde 3-phosphate dehydrogenase (GAPDH) expression was measured as a reference. PCR was conducted using the following cycling conditions: pre-incubation and denaturation at 95 °C during 10 min. Amplification with 35 or 40 cycles that included: denaturation at 95 °C for 10 s with a thermal ramp rate at 20 °C/s; annealing at 61 °C for 7 s with a thermal ramp rate at 20 °C/s; amplification at 72 °C for 10 s with a thermal ramp rate at 20 °C/s using GAPDH primers. The primer design for the selected genes for RT-qPCR analysis was performed using the Primer-BLAST bioinformatics tool (Table S1).

### KEGG pathway enrichment

The KEGG enrichment analysis was conducted using WebGestalt (Zhang, Kirov & Snoddy, 2005). To this end, the 5% most expressed genes shared between the 16 arrays were selected to identify the KEGG pathways significantly enriched. Thus, the pathway enrichment was conducted using 1,539 genes. All human genes were used as background to calculate statistical significance and setting the hypergeometric test for enrichment evaluation analysis. The *p* values were adjusted using the Benjamini–Hochberg multiple test adjustment. Finally, we identified the enriched KEGG pathways with an adjusted *P*-value (*p* ≤ e^−9^).

**Figure 1:**
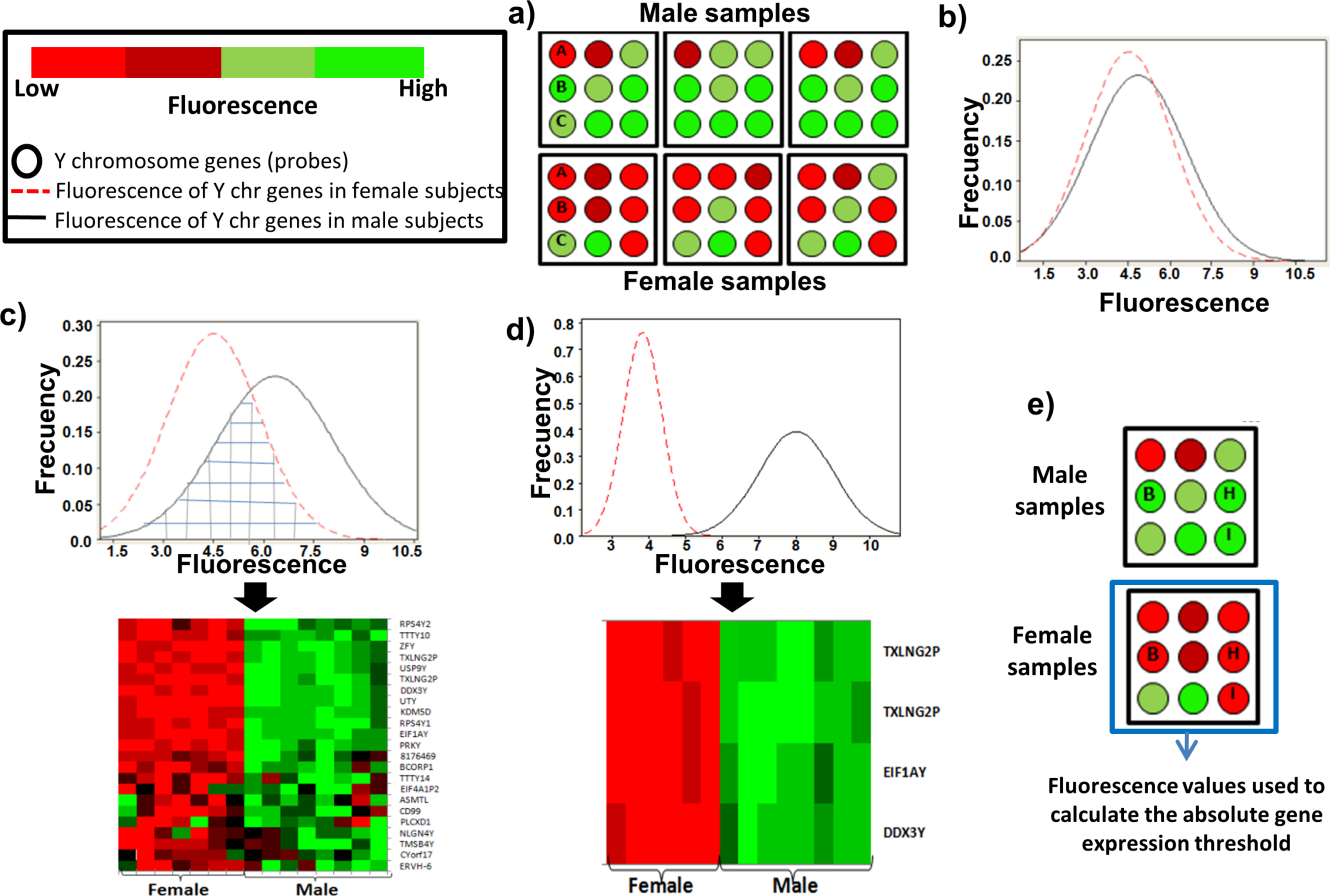
Determination of a threshold to measure the absolute gene expression based in the Y chromosome genes. (A) Schematic representation of Y-chromosome genes with similar fluorescence intensity between female and male subjects (gene A or C), and genes with sex-dependent fluorescence (gene B). (B) Histogram of fluorescence for the 124 Y-chromosome genes in males (black line) and females subjects (dotted red line). (C) Histogram of fluorescence for the 23 Y-chromosome genes that showed a statistically significant sex-dependent fluorescence. The gridded region showed the fluorescence of the genes overlapped between male and female. The heatmap illustrates the real fluorescence level for these genes in our leukocyte samples. (D) Histogram of fluorescence distribution of the three genes (four probes) used to calculate the absolute gene expression threshold. Their fluorescence does not overlap between male and female subjects. The heatmap illustrates the real fluorescence level for these four probes in our leukocyte samples. (E) Schematic representation of the three genes (four probes) used to calculate the absolute gene expression threshold for the microarray.

## Results

### Selection of the Y chromosome genes to establish the absolute gene expression threshold

The Y chromosome is the sex-determining genetic element in higher mammals, and it is absent in female subjects. Therefore, the fluorescence of the Y chromosome genes in female subjects could be used as the background fluorescence for all the probes in the microarray. To test this hypothesis, the transcriptome of leukocytes from 16 human samples were analyzed. After microarray data normalization, we obtained the fluorescence intensities for the 124 genes of the Y chromosome (Table S2). We found several genes with similar fluorescence intensity between male and female subjects (gene A and gene C in Fig. 1A), while other genes had a sex-dependent differential fluorescence (gene B in Fig. 1A). The histogram with the fluorescence of the 124 genes did not show a differential fluorescence distribution between male and female subjects (Fig. 1B). To discern the genes with a sex-dependent expression, only the genes with a significant difference (*p* ≤ 0.05) in fluorescence between the sex groups were selected (Fig. 1C). However, several genes showed an overlapped fluorescence between male and female (gridded region in Fig. 1C). Thus, we eliminated the genes which fluorescence distribution was overlapped between sexes. After that, only three genes named DDX3Y (probe: 8176624), EIF1AY (probe: 8176719), and TXLNG2P (probes: 8176709 and 8176698) showed a fluorescence significantly different between sexes (*p* = 4.10^−32^) (Fig. 1D, Table S3).

**Figure 2:**
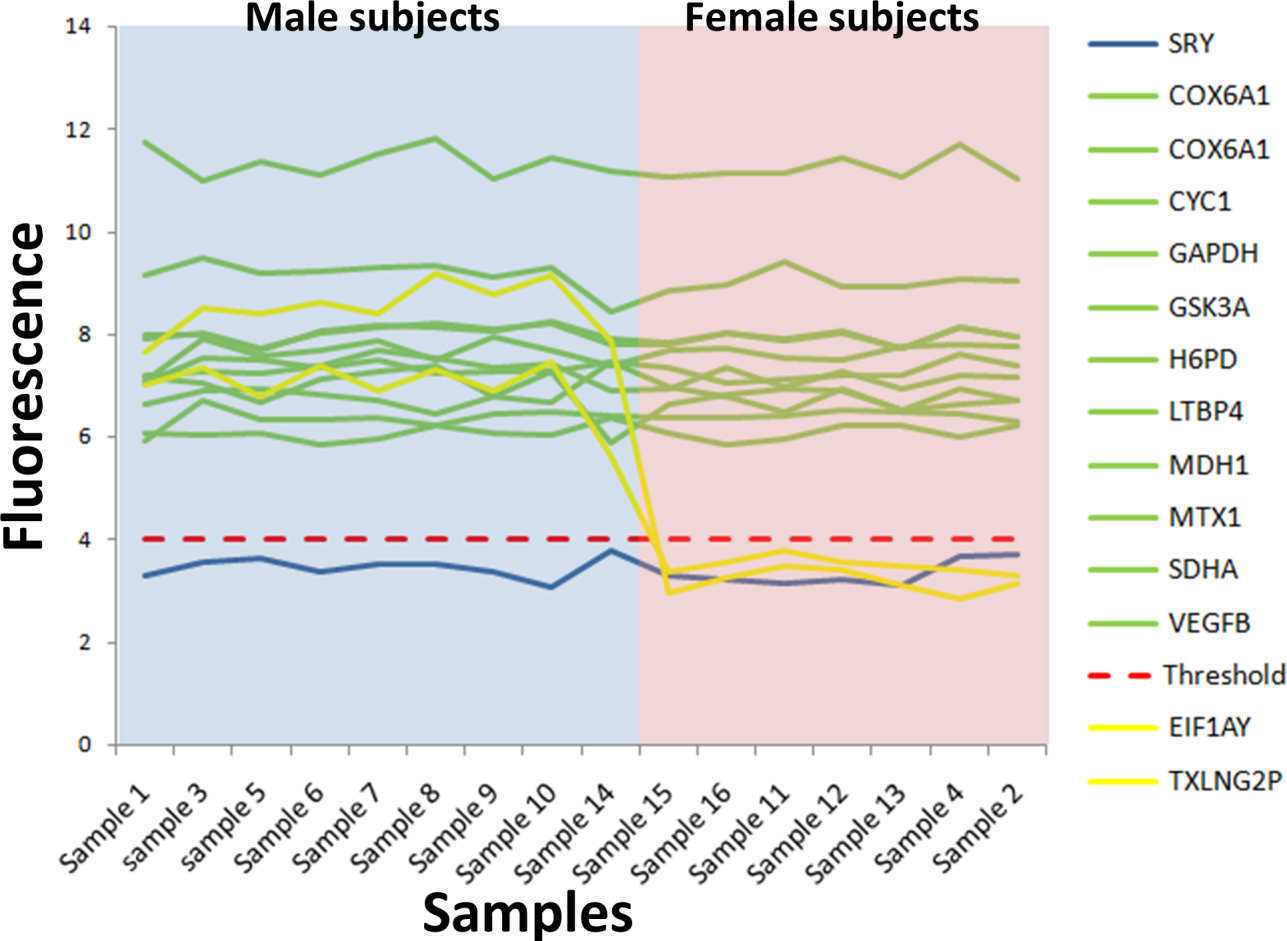
Microarray fluorescence of the housekeeping and SRY genes. The microarrays from male and female subjects are in blue and pink areas, respectively. The fluorescence of the housekeeping genes (green lines) was above the absolute gene expression threshold (dotted red line), while the fluorescence level of the SRY gene was under the absolute gene expression threshold (blue line). Two Y chromosome genes used to calculate the expression threshold were illustrated in orange lines. The microarray identifier corresponding to each sample number is shown in Table S8.

Thus, the fluorescence emitted by these three genes in female subjects (Fig. 1E) was used to calculate the upper limit of fluorescence with a 95% confidence. The value found was 4 and it was selected as the absolute gene expression threshold. Hence, the genes with fluorescence values ≥4 were considered as expressed, while the genes with fluorescence <4 were considered as non-expressed genes (background fluorescence).

We selected 11 housekeeping genes from the Cheng-Wei (2011) study (Chang et al., 2011) and their fluorescence intensity (Fi) was analyzed in our 16 microarray samples. These genes have high (>10), medium (6–10)or low (4–6)fluorescence intensity (Fig. 2, Table S4) and according to the absolute gene expression threshold (≥4), the 11 housekeeping genes were expressed in all samples (green lines in Fig. 2). Importantly, their fluorescence was not sex-dependent (*p* = 0.81) (blue and pink zones in Fig. 2). This result was expected because housekeeping genes are typically expressed under normal conditions. Also, there were housekeeping genes with high and low fluorescence values as compared to the fluorescence of the absolute gene expression threshold (red dotted line in Fig. 2).

The SRY gene was analyzed as a negative control of the gene expression. We found that SRY fluorescence was under the absolute gene expression threshold, indicating that this gene was not expressed in either sample (blue line in Fig. 2). Furthermore, the fluorescence of the SRY gene was not significantly different between sexes (*p* = 0.93) (blue line in Fig. 2). This result was expected because it has been reported that SRY gene, unique for the Y chromosome, is only expressed during the human embryonic development in male subjects (Berta et al., 1990).

**Figure 3:**
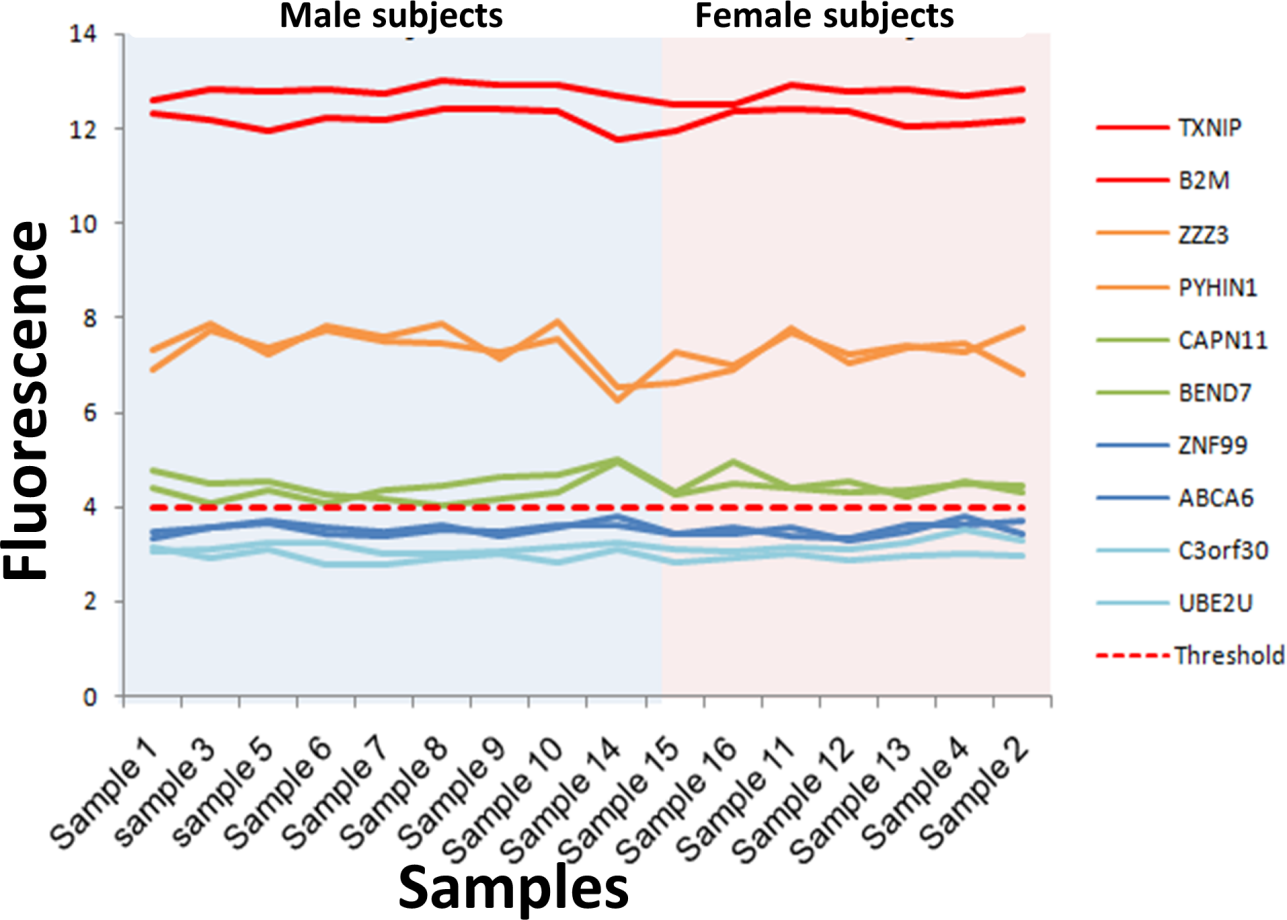
Microarray fluorescence of the genes selected for RT-qPCR. The fluorescence of the 10 genes selected for RT-qPCR analysis is shown for male (blue area) and female (pink area). The genes with fluorescence above the absolute gene expression threshold is shown in green, orange and red lines, while the fluorescence of genes under the threshold is shown in blue lines.

### Validation of the absolute gene expression threshold by RT-qPCR

**Figure 4:**
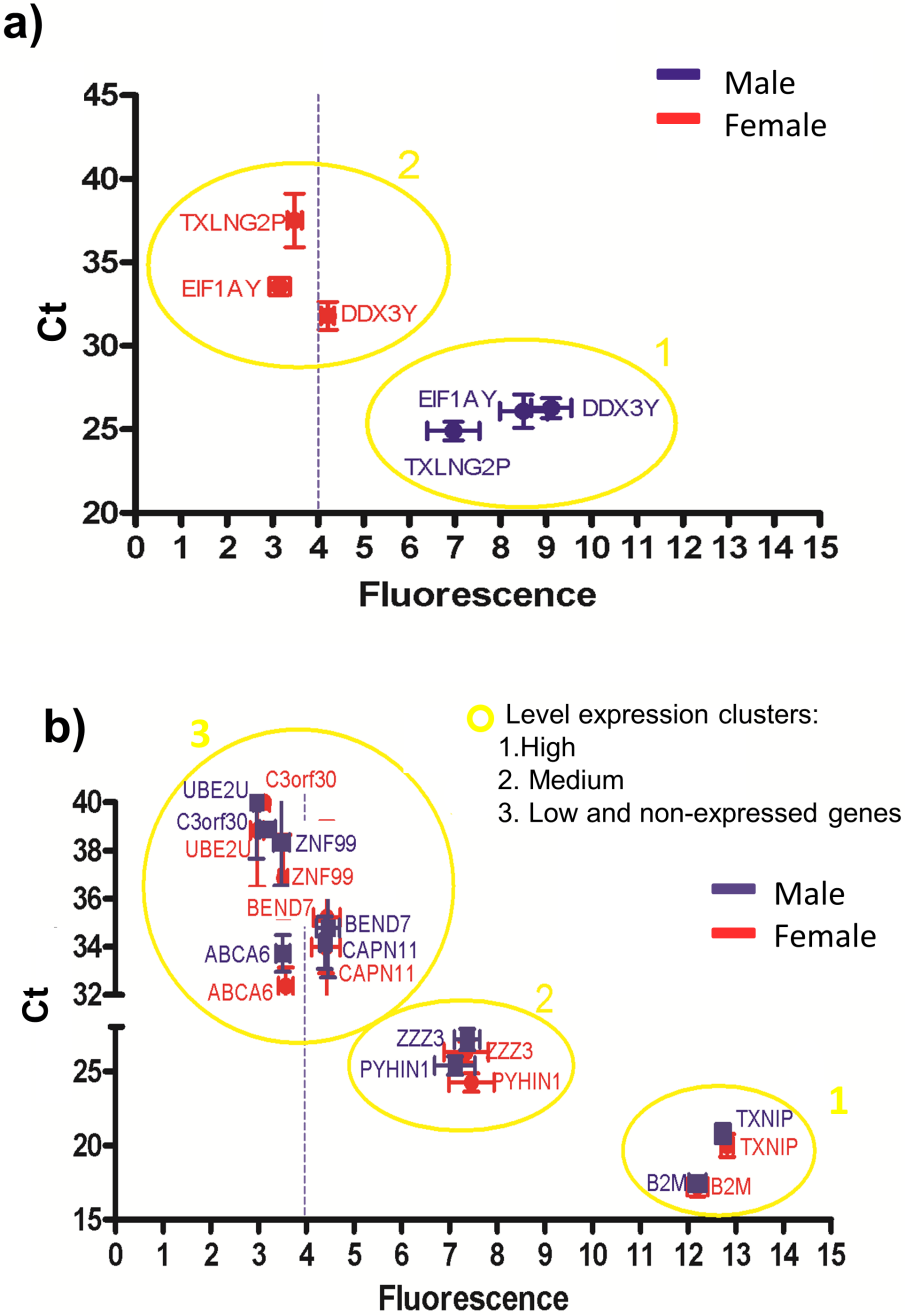
Correlations between the gene expression levels obtained by RT-qPCR (Ct values) and microarray (fluorescence values). The blue and red symbols represent male and female samples, respectively. The absolute gene expression threshold (Fi = 4) is represented as a blue dotted line. (A) Genes used to calculate the absolute gene expression threshold. (B) Genes with different fluorescence values (low to high). According to the Ct values and fluorescence levels three clusters were showed: highly expressed genes clustered in group 1, medium expressed genes clustered in group 2, and low expressed genes clustered with the non-expressed genes in group 3.

The consistency of the absolute gene expression threshold in assessing the expression of a gene was also evaluated using RT-qPCR. To this end, we selected ten genes that exhibited the lowest variability of fluorescence intensity in all the microarrays, and their expression levels were measured by RT-qPCR. These genes were separated into three groups according to their fluorescence level (Fig. 3, Table S5). The first group contained six expressed genes with fluorescence intensity above the absolute gene expression threshold (Fi ≥ 4). This group included two genes with high fluorescence intensity (Fi ≥ 10, red lines in Fig. 3), two genes with medium fluorescence intensity (Fi 6–10, orange lines in Fig. 3) and two genes with low fluorescence intensity (Fi 4–6, green lines in Fig. 3). The second group contained a set of non-expressed genes (fluorescence intensities below the absolute gene expression threshold of 4). This second group included two genes with fluorescence close to the expression threshold (3–4, dark blue lines in Fig. 3) and two genes with the lowest fluorescence (Fi < 3, light blue in Fig. 3). The third group contained the three genes used to define the absolute gene expression threshold (DDX3Y, EIF1AY and TXLNG2P) by RT-qPCR. Importantly, the expression level observed between RT-qPCR (measured by Ct: Ct threshold cycle) and microarray (measured by fluorescence intensity) had a good Spearman correlation (*r* = 0.882, *p* = 2.84^−69^) on the 13 genes (Fig. S1). The fluorescence of the genes DDX3Y, EIF1AY and TXLNG2P shown a significant difference between male and female subjects (*p* = 6.74^−20^) (Fig. 4A, Table S6), while, the fluorescence level of the other 10 genes was not significantly different between sexes (*p* = 0.98) (Table S7).

According to the Ct and fluorescence levels (Fig. 4B), three clusters of genes were observed: one cluster containing highly expressed genes (cluster 1), another cluster containing medium expressed genes (cluster 2) and a third cluster containing low and the non-expressed genes (cluster 3). These results suggest that the absolute gene expression threshold not only assessed what genes are expressed, but also allowed the level of expression according to the fluorescence intensity. However, the genes BEND7 and CAPN11 have low expression and their fluorescence values overlapped with the non-expressed genes, suggesting that genes with low fluorescence (close to the absolute gene expression threshold) can be really not expressed genes (cluster 3 in Fig. 4B).

### Human transcriptome profiling using the most expressed genes

We selected the 5% of genes with the highest fluorescence intensity (most expressed genes) shared between the 16 microarrays to identify the KEGG pathways significantly enriched, resulting in 1,539 genes that were expressed in all the leukocyte samples. The pathway enrichment analysis showed 45 significantly enriched pathways (*p* ≤ E−9) (Table S9). Interestingly, the most enriched pathways were those expected for a leukocyte cell type (Table 1). For example, Phagosome (*p* = 5.67E−35), chemokine signaling pathway (*p* = 1.62E−21), Fc gamma R-mediated phagocytosis (*p* = 6.49E−23), Natural Killer cell mediated cytotoxicity (*p* = 1.5E−19), antigen processing and presentation (*p* = 9.22E−24), T cell receptor signaling pathway (*p* = 9.08E−27), B cell receptor signaling pathway (*p* = 5.77E−14) and leukocyte transendothelial migration (*p* = 5.74E−17).

**Table 1:** Top 20 enriched KEGG pathways in human leukocyte transcriptomes.

**Ranking**	**Pathway name**	**# Gene**	***p* value**
1	Ribosome	61	7.62E−64
2	Phagosome	52	5.67E−35
3	Regulation of actin cytoskeleton	54	3.43E−29
4	Antigen processing and presentation	31	9.22E−24
5	Fc gamma R-mediated phagocytosis	33	6.49E−23
6	Leishmaniasis	29	3.37E−22
7	Chemokine signaling pathway	43	1.62E−21
8	Osteoclast differentiation	36	1.92E−21
9	Protein processing in endoplasmic reticulum	40	3.21E−21
10	Natural killer cell mediated cytotoxicity	35	1.5E−19
11	Endocytosis	41	7.79E−19
12	Pathways in cancer	52	8.53E−19
13	Leukocyte transendothelial migration	30	5.74E−17
14	Focal adhesion	38	1.76E−16
15	Hematopoietic cell lineage	26	2.24E−16
16	Viral myocarditis	23	1.05E−15
17	Shigellosis	21	7.19E−15
18	Pathogenic Escherichia coli infection	20	1.45E−14
19	Toxoplasmosis	29	1.57E−14
20	B cell receptor signaling pathway	22	5.77E−14

### Application of the absolute gene expression threshold to another microarray platform

We calculated the absolute gene expression threshold for previously published microarray data of liver cells (GSE18269) using our approach (Fig. 1). After that, we selected the 5% most expressed genes shared between all samples, resulting in a set of 1,547 expressed genes which were used to identify the significantly enriched pathways. We found that 37 KEGG pathways (*p* ≤ E−9) were significantly enriched (Table S10). The majority of these enriched pathways were functional related with liver cells (Table 2). For example, Complement and coagulation cascades (*p* = 9.19E−37), Drug metabolism-cytochrome P450 (*p* = 3.5E−24), Fatty acid metabolism (6.2E−22), Metabolism of xenobiotics by cytochrome P450 (8.03E−21) and Glycolysis/Gluconeogenesis (1.8E−19).

**Table 2:** Top 20 enriched KEGG pathways in human liver cells transcriptomes.

**Ranking**	**Pathway name**	**# Gene**	***p* value**
1	Metabolic pathways	266	2.57E−148
2	Ribosome	76	4.39E−95
3	Oxidative phosphorylation	60	4.07E−51
4	Parkinson’s disease	59	2.5E−50
5	Huntington’s disease	64	4.26E−46
6	Protein processing in endoplasmic reticulum	60	1.97E−44
7	Alzheimer’s disease	58	1.1E−41
8	Complement and coagulation cascades	38	9.19E−37
9	Drug metabolism—cytochrome P450	30	3.5E−24
10	Fatty acid metabolism	23	6.2E−22
11	Valine, leucine and isoleucine degradation	23	1.15E−21
12	Metabolism of xenobiotics by cytochrome P450	27	8.03E−21
13	Glycolysis/Gluconeogenesis	25	1.8E−19
14	Protein export	16	4.54E−18
15	Arginine and proline metabolism	22	7.13E−18
16	Peroxisome	25	3.6E−17
17	Glycine, serine and threonine metabolism	17	2.19E−16
18	Retinol metabolism	22	4.47E−16
19	PPAR signaling pathway	22	3.75E−15
20	Propanoate metabolism	16	5.9E−15

Additionally, we calculated the absolute gene expression threshold for another microarray platform. To this end, we selected the previously reported GSE22974 data in which yeast RNA was hybridized to a human microarray (U133 plus 2.0) and the absolute gene expression threshold was calculated for this dataset. After that, the fluorescence value for the absolute gene expression threshold was 9.17. Thus, all the probes with fluorescence above this fluorescence value were considered as expressed. According to our expression threshold, the 97.9% (*n* = 53,466) of the probes were not expressed (Fig. S2). This result was expected, because the hybridized RNA was from yeast and the probes on the microarray were designed for human genes.

The Gene Expression Barcode algorithm provides absolute measures of expression for human genes using an expression threshold for each probe according to the probe effect of each one (McCall et al., 2013). We applied this algorithm on the same data (GSE22974) to compare their results with the obtained using our expression threshold. The Barcode algorithm found that 94% (*n* = 51,196) of probes also were not expressed. Importantly, the 93.7% (50,638) of these non-expressed probes were shared between Barcode and our approach (Fig. S3). These results suggest a good consistency to call expressed genes between the Barcode algorithm and the threshold approach reported here.

## Discussion

In this work, we demonstrated the utility of a novel method to measure the absolute gene expression of microarray data based on the fluorescence of the Y chromosome genes. The Y chromosome is unique in male subjects; however, we determined that many Y chromosome genes had similar gene expressions between sexes, probably because 95% of the Y chromosome genes are encoded in the non-recombining region (NRY), where many genes have homologs in the X chromosome, suggesting that the expression of these genes could be similar between male and female. Additionally, other genes are encoded in the pseudoautosomal region (PAR) of the Y chromosome, which exchanges genetic material with the pseudoautosomal region of the X chromosome during meiosis, therefore, PAR genes are identical on X and Y chromosomes (Helena Mangs & Morris, 2007). In consequence, the expression levels for PAR genes could be similar between male and female samples.

The expression of DDX3Y, EIF1AY and TXLNG2P proved to be sex-dependent; therefore, these genes were used to calculate the absolute gene expression threshold. These three genes are located in a locus called AZoospermia Factor (AZF), which is associated with spermatogenesis (Quintana-Murci & Fellous, 2001; Kleiman et al., 2007). AZF encodes the non-recombining region of the human Y chromosome (NRY) that does not cross over with the X chromosome (Quintana-Murci & Fellous, 2001), suggesting a sex-specific expression of the three genes. Although, the DDX3Y and EIF1AY genes also have X-linked homologous genes (Yang et al., 2006). Interestingly, it has been reported that these two genes also can be expressed in blood cells that encode for human male-specific minor histocompatibility antigens which contribute to immune-mediated disease (Miklos et al., 2005), suggesting that their expression could be sex-specific.

Our results showed a strong correlation among the gene expression measured by absolute gene expression threshold and RT-qPCR (Figs. 4A and 4B). Additionally, the fluorescence level for each gene on the microarray correlated with the values of gene expression by RT-qPCR (Figs. 2 and 3) and the KEGG pathway analysis shows that the profile of each cell is still clearly established and not affected by our method. Moreover, the SRY gene was not expressed in any of the samples, correlating with the fact that SRY gene only is expressed during the male embryonic development (Berta et al., 1990). However, when establishing the level of expression, the RT-qPCR analysis suggested that genes with fluorescence values close to the absolute gene expression threshold could also be not expressed genes (cluster 3 in Fig. 4B). This effect may be related to the fact that our absolute gene expression threshold is a general value for all the probes in the microarray and it does not identify the background fluorescence noise and the dynamic range of fluorescence per probe. Nonetheless, our results suggest that the threshold could be successfully used to determine the expressed genes with medium and high fluorescence intensities.

We suggest that the absolute gene expression threshold could be applied in any microarray platform of gene expression for human given that the biology of the Y-chromosome genes is independent of the microarray technology. Plus, the application of threshold to measure the transcriptome profiling of liver cells demonstrated that our method can also be applied to different cell types. On the other hand, the yeast RNA hybridized in a different human transcriptome array (U133 Plus 2.0) showed that 97.9% of the probes were not expressed. Similar results were confirmed when we applied the Barcode algorithm to the same dataset (Figs. S2 and S3). These results suggest that the threshold can be used in other microarray platforms of human gene expression. However, a limitation of our method is that microarrays with female samples are always required to calculate the absolute gene expression threshold of the experiment. Thus, each absolute gene expression threshold should be project-specific and is difficult to generalize this value. In addition, the application of our method on microarrays from subjects with Y or X chromosome diseases is not recommended, because the determination of the fluorescence threshold could be skewed.

The Gene Expression Barcode algorithm computes a gene expression threshold and it only evaluates if a gene is or not expressed (McCall et al., 2013). In contrast, our method allows to measure the level of expression based in fluorescence values, therefore, it is possible not only to assess the absence or presence calls of a transcript, but also allows to quantify the expression level according to the observed fluorescence. In addition, our threshold allows grouping the expression of each gene in low, medium and highly expressed gene. Another important web tool to infer the absolute gene expression of genes is Gene Expression Commons, which is based on meta-analysis of large-scale microarray data (Seita et al., 2012). However, this platform focuses on gene expression profiling of the hematopoietic system. In contrast, our threshold can be applicable to any human cell type due that Y chromosome genes are generally ubiquitously expressed (Maan et al., 2017). Therefore, we believe that our threshold serves as a common method for profiling gene expression in any human cell type.

Several Affymetrix microarrays contain approximately twenty probes for the same RNA probe target. Half of these are “mismatch probes”, which do not match the target sequence and they are used to measure the amount of nonspecific binding by the MAS5 algorithm. In this manner the user can obtain if the gene is present or absent. However, the Affymetrix Gene Chip Human Gene 1.0 ST Array that we used does not have mismatch probes; therefore, MAS5 cannot be used to define a probe set as present or absent call. Thus, the conventional background correction approach is not applicable for this array, increasing the need of tools as our threshold approach to predict present or absent calls of gene expression.

## Conclusions

The measurement of the absolute gene expression eliminates the need of collecting control samples to perform a reference array. Thus, we presented a novel approach to measure the absolute gene expression from microarray data of any cell type, giving the transcriptome profiling of leukocyte cell type as an example of the application of this method. There are many databases like Oncomine  (Rhodes et al., 2004), QIAGEN’s Ingenuity®, Flymine (Lyne et al., 2007), TiGER (Liu et al., 2008), BODYMAP (Hishiki et al., 2000) and BioGPS (Wu et al., 2016) that provide comprehensive data from microarray samples. However, none of these databases focuses on absolute measurements of gene expression. We consider that our approach constitutes a novel method to estimate the present or absent transcripts and the level of expression from a microarray, complementing the methods that currently exist to measure the absolute gene expression such as Gene Expression Barcode (McCall et al., 2013) and Gene Expression Commons (Seita et al., 2012).

##  Supplemental Information

10.7717/peerj.4133/supp-1 Table S1 List of primers used for gene expression analysis by RT-qPCR For each gene listed in the first column, the forward (Fwd) and reverse (Rev) primer sequences are shown as well as the product length.

10.7717/peerj.4133/supp-2 Table S2 Fluorescence obtained for the 124 genes of the Y chromosome The fluorescence intensities for 124 genes of the Y chromosome were obtained from 16 human leukocyte samples (9 males and 7 female subjects) using the HuGene 1.0 ST microarray of Affymetrix (GSE89571). In the first column the Y chromosome genes are numbered. The names of the Y chromosome genes are listed in the second column. The third column mentions the probe codes for each gene. Some Y chromosome genes have two or more probes and in the following columns, the first nine arrays correspond to male samples and the last seven arrays correspond to female samples.

10.7717/peerj.4133/supp-3 Table S3 Fluorescence obtained for the 3 genes of the Y chromosome selected for absolute gene expression threshold Fluorescence intensities obtained for DDX3Y, EIF1AY and TXLNG2P genes in male (M) and female (F) subjects using the Affymetrix HuGene 1.0 ST microarray (GSE89571).

10.7717/peerj.4133/supp-4 Table S4 Fluorescence obtained for housekeeping genes and the SRY gene The fluorescence intensities of the genes were obtained by the HuGene 1.0 ST microarray of Affymetrix (GSE89571) for male (M) and female (F) samples.

10.7717/peerj.4133/supp-5 Table S5 Fluorescence obtained in the microarray for the 13 genes that were analyzed using RT-qPCR

10.7717/peerj.4133/supp-6 Table S6 Y chromosome gene expression levels obtained by microarray and Real Time PCR Gene expression data were normalized according to the reference gene (Glyceraldehyde 3-phosphate dehydrogenase (GAPDH)). The ID samples are listed in the first column. Cp, crossing point; RelExp, relative expression.

10.7717/peerj.4133/supp-7 Table S7 Gene expression levels obtained by Real Time PCR and microarray Gene expression data were normalized according to the reference gene (Glyceraldehyde 3-phosphate dehydrogenase (GAPDH)). The ID samples are listed in the first column. Cp, crossing point; RelExp, relative expression.

10.7717/peerj.4133/supp-8 Table S8 ID sample or array (right column) corresponding to each sample number (left column) in main figures

10.7717/peerj.4133/supp-9 Table S9 Enriched KEGG pathway analysis in human leukocytes transcriptomes Complete list of significant enriched KEGG pathways (*p* ≤ E–9) obtained after analyzing the total 5% most expressed leukocyte genes of 16 subjects.

10.7717/peerj.4133/supp-10 Table S10 Enriched KEGG pathway analysis in human liver cells transcriptomes Complete list of significant enriched KEGG pathways (*p* ≤ E–9) obtained after analyzing the total 5% most expressed genes liver cells arrays in GSE18269.

10.7717/peerj.4133/supp-11 Figure S1 Correlation between microarray and RT-qPCR data The expression values of 13 different genes are showed in the graph. The expression values by microarrays (fluorescence) are represented in the *X* axis, and the expression values by RT-qPCR (Cp Values) are represented on the *Y* axis. The Spearman correlation was *r* =  − 0.882, *p* = 2.84–69.

10.7717/peerj.4133/supp-12 Figure S2 Histogram of the fluorescence intensities of a microarray with human probes (U133 plus 2.0 of Affymetrix) in which yeast RNA was hybridized (GSE22974) The distribution of the fluorescence intensities are showed and the fluorescence value for the absolute gene expression threshold is marked by the vertical line in graph (absolute gene expression threshold = 9.17). All probes with fluorescence under this value were considered as not-expressed (97.9% (*n* = 53,466)). The raw data of fluorescence was converted in log2 to obtain a clearer histogram.

10.7717/peerj.4133/supp-13 Figure S3 Venn diagram about the non-expressed probes of a human microarray (U133 plus 2.0 of Affymetrix) in which yeast RNA was hybridized (GSE22974) In the left circle, we showed the number (558) and percent (1%) of probes that are not expressed by Barcode (Shyamsundar et al., 2005; Lipshutz et al., 1999; Tang et al., 2007) and are different to the non-expressed probes by the absolute gene expression threshold. In the second circle and on the right side is showing the number (2828) and the percent (5%) of probes that are not expressed by the absolute gene expression threshold and are different to the non-expressed probes by Barcode (Shyamsundar et al., 2005; Lipshutz et al., 1999; Tang et al., 2007). In the middle of both circles are the number (50638) and percent (93.7%) of the probes that are non-expressed by both Barcode and the absolute gene expression threshold.

10.7717/peerj.4133/supp-14 Supplemental Information 1 Miame checklist

## Additional Information and Declarations

### Data Availability

The following information was supplied regarding data availability: The raw expression data is available on the NCBI Gene Expression Omnibus (GEO) under accession number GSE89571.
