# Supplementary material for: A novel approach for human whole transcriptome analysis based on absolute gene expression of microarray data
Source: PeerJ. 2017 Dec 8;5:e4133. doi: 10.7717/peerj.4133 (PMC5724404; doi:10.7717/peerj.4133)
Supplement: Table S1 — For each gene listed in the first column, the forward (Fwd) and reverse (Rev) primer sequences are shown as well as the product length. [file peerj-05-4133-s001.pdf]

Gene	Primer	Sequence (5´-> 3´)	Amplicon Length
**DDX3Y**	Fwd	AGGACGTGTAGGAAACCTGG	111 bp
	Rev	GAAGGCACTTCTTGTTTAGCTTC	
**EIF1AY**	Fwd	ACAATGCAGATGAAGCTAGAAGC	100 bp
	Rev	TCATCATCATCTCCAGGACCAA	
**TXLNG2P**	Fwd	AAAGAACAGGAGTTGGGTGAGC	100 bp
	Rev	TAAAGCTTGCTGACCAGACGG	
**TXNIP**	Fwd	AGTAGTGGATCTGGTGGATGTC	100 bp
	Rev	CCGCCCATCAGGAATGAACA	
**B2M**	Fwd	GAGATGTCTCGCTCCGTGG	111 bp
	Rev	CTCTGCTGGATGACGTGAGT	
**PYHIN1**	Fwd	CACTGGGAGACCTTGCTGAA	110 bp
	Rev	GCATGCAGGTGTAGCAGGAT	
**ZZZ3**	Fwd	GGCACCAGAGAGAAGCACTG	120 bp
	Rev	GGTCCCATACGATCTCAGGC	
**BEND7**	Fwd	CCTGGTGAAGCTGACAGACA	118 bp
	Rev	TCCTTCTTTGCCAACTCGCT	
**CAPN11**	Fwd	CAAGAGCTACTGGCACACCA	107 bp
	Rev	TAAACTGGGGGTTGGTCCAG	
**ZNF99**	Fwd	GCAATGCCTGGACATGGCT	103 bp
	Rev	CAAGTCTAGCTTAGAGACAGCGA	
**ABCA6**	Fwd	AAAGCGTGTATCAGCAAACCA	100 bp
	Rev	AGTATTGAGAGGCCCCATTCC	
**UBE2U**	Fwd	ACTATGCTCCTCCAGTTGTGA	107 bp
	Rev	CCACTTCTCAGGGTTGTCCA	
**C3ORF30**	Fwd	CCCAAGACTCCCCTCCATCT	113 bp
	Rev	AACCCTTTCCTTGGTCAATTTCTG	
