# Supplementary material for: A novel approach for human whole transcriptome analysis based on absolute gene expression of microarray data
Source: PeerJ. 2017 Dec 8;5:e4133. doi: 10.7717/peerj.4133 (PMC5724404; doi:10.7717/peerj.4133)
Supplement: Table S2 — The fluorescence intensities for 124 genes of the Y chromosome were obtained from 16 human leukocyte samples (9 males and 7 female subjects) using the HuGene 1.0 ST microarray of Affymetrix (GSE89571). In the first column the Y chromosome genes are numbered. The names of the Y chromosome genes are listed in the second column. The third column mentions the probe codes for each gene. Some Y chromosome genes have two or more probes and in the following columns, the first nine arrays correspond to male samples and the last seven arrays correspond to female samples. [file peerj-05-4133-s002.pdf]

	Gene Name	Probesets	NI0627.CEL	VE9-0291.CEL	VE9-0336.CEL	VE9-0432.CEL	VE9-0472.CEL	VE9-0515.CEL	VE9-0567.CEL	VE9-0687.CEL	VE9-0817.CEL	VE9-1036.CEL	VE9-1050.CEL	VE9-0697.CEL	VE9-0739.CEL	VE9-0748.CEL	VE9-0307.CEL	VE9-0039.CEL
1	AKAP17A	8176342	6.03	6.15	6.11	5.93	5.88	6.00	6.22	6.42	6.16	6.14	6.22	6.06	6.09	5.98	6.06	6.16
2	AMELY	8177061	4.30	4.11	4.10	4.23	4.22	3.98	4.13	3.86	4.55	4.14	4.01	4.20	4.21	4.04	4.38	4.09
3	ANKRD36P1	8176951	3.78	4.13	4.27	4.40	4.14	4.34	4.06	4.04	4.38	4.10	3.94	4.23	4.25	4.40	4.26	4.29
4	ASMTL	8177011	6.06	6.52	6.35	6.35	6.51	6.27	6.48	6.58	6.47	5.81	5.96	6.24	6.26	6.24	6.61	6.10
5	ASMTL-AS1	8176336	5.65	5.82	5.99	5.89	5.46	5.53	5.91	5.88	6.25	5.55	5.70	5.49	5.91	5.69	6.05	6.05
6	BCORP1	8177229	3.82	6.04	5.27	5.01	5.17	5.03	4.83	5.60	4.83	3.04	3.69	3.42	3.38	3.56	2.82	2.90
7	BPY2	8176821	5.92	4.11	4.17	3.92	3.93	3.93	3.94	3.72	4.45	4.18	3.79	3.62	3.87	3.82	4.06	3.90
		8176867	5.92	4.11	4.17	3.92	3.93	3.93	3.94	3.72	4.45	4.18	3.79	3.62	3.87	3.82	4.06	3.90
		8177449	5.92	4.11	4.17	3.92	3.93	3.93	3.94	3.72	4.45	4.18	3.79	3.62	3.87	3.82	4.06	3.90
8	CD24	8177222	4.82	6.32	5.52	6.16	6.28	5.85	5.98	6.32	4.62	5.09	5.98	4.78	5.71	5.81	5.30	5.49
9	CD99	8176360	9.09	9.45	9.17	9.36	9.62	9.71	9.16	9.42	8.83	8.45	8.73	9.04	9.03	8.68	9.42	8.68
10	CDY1	8177186	4.03	3.37	3.72	3.48	3.71	3.61	3.84	3.57	3.86	3.42	3.64	3.97	3.81	3.67	3.91	3.66
		8176671	4.22	3.50	3.87	3.57	3.84	3.72	3.97	3.69	4.01	3.56	3.77	4.06	3.96	3.77	4.03	3.78
11	CDY2A	8177405	4.18	3.49	3.70	3.50	3.75	3.58	3.78	3.67	4.04	3.52	3.74	3.98	3.87	3.62	3.91	3.64
		8176926	4.18	3.49	3.70	3.50	3.75	3.58	3.78	3.67	4.04	3.52	3.74	3.98	3.87	3.62	3.91	3.64
12	CSF2RA	8176306	7.59	7.42	7.25	7.58	7.86	8.32	8.13	8.34	7.67	7.41	8.19	7.78	8.01	7.98	8.03	7.28
13	CSPG4P1Y	8176923	5.52	4.62	5.22	4.84	4.93	4.89	4.53	4.62	5.71	4.73	4.67	4.59	4.90	4.99	4.99	4.61
		8177410	5.52	4.62	5.22	4.84	4.93	4.89	4.53	4.62	5.71	4.73	4.67	4.59	4.90	4.99	4.99	4.61
14	CYorf17	8177273	5.29	5.29	5.20	5.04	5.12	5.07	4.91	5.06	6.06	4.90	4.86	4.57	5.22	4.87	5.06	4.91
15	DAZ4	8177354	5.25	3.87	4.00	3.78	3.86	3.80	3.97	3.75	4.46	3.71	3.91	3.58	3.92	3.89	3.83	3.50
		8176832	5.25	3.87	4.00	3.78	3.86	3.80	3.97	3.75	4.46	3.71	3.91	3.58	3.92	3.89	3.83	3.50
		8176878	5.25	3.87	4.00	3.78	3.86	3.80	3.97	3.75	4.46	3.71	3.91	3.58	3.92	3.89	3.83	3.50
		8177426	5.26	3.93	4.09	3.87	3.95	3.89	4.05	3.80	4.53	3.80	3.99	3.69	4.02	4.00	3.88	3.55
16	DDX3Y	8176624	8.81	9.19	9.10	9.44	9.27	9.51	9.16	9.46	8.09	4.21	4.13	4.24	4.15	4.30	3.99	4.45
17	DUX4L7	8176570	8.29	6.67	6.80	6.50	6.19	6.50	5.94	6.00	7.30	7.47	6.84	6.20	6.88	6.24	5.51	6.50
18	EIF1AY	8176719	7.65	8.51	8.39	8.63	8.40	9.19	8.79	9.14	7.86	2.83	3.14	3.46	3.37	3.11	3.25	2.93
19	EIF4A1P2	8177046	4.85	5.68	5.14	5.31	5.16	5.60	5.56	5.42	5.04	5.04	5.30	5.06	5.31	4.56	4.54	4.11
20	ERVH-6	8177269	4.75	4.52	3.78	4.48	4.33	4.28	4.17	3.79	4.39	3.95	3.64	3.47	3.61	3.69	4.09	3.45
21	FLJ20518	8176572	7.82	5.69	5.31	5.26	4.67	5.01	5.38	5.25	5.99	6.62	5.85	5.39	6.29	5.24	5.32	5.34
22	GOLGA2P2Y	8177413	6.70	5.91	6.07	5.78	5.91	5.69	6.07	5.72	6.50	5.84	6.20	5.76	5.92	6.11	5.94	5.84
		8176910	6.70	5.91	6.07	5.78	5.91	5.69	6.07	5.72	6.50	5.84	6.20	5.76	5.92	6.11	5.94	5.84
23	HSFY2	8177201	2.97	3.03	3.16	2.84	2.91	2.86	3.21	2.86	3.25	3.19	3.19	2.96	2.95	3.11	3.23	3.13
		8176681	2.97	3.03	3.16	2.84	2.91	2.86	3.21	2.86	3.25	3.19	3.19	2.96	2.95	3.11	3.23	3.13
24	IL3RA	8176323	5.27	5.98	5.76	5.76	6.01	6.10	6.15	6.06	5.63	5.29	5.42	6.03	5.76	5.63	6.60	5.71
25	IL9R	8176972	6.42	5.93	6.29	5.96	6.05	6.03	5.95	5.87	6.61	6.23	6.01	5.84	6.05	5.96	6.00	5.78
26	KDM5D	8177232	8.11	8.55	8.20	8.25	8.25	8.30	8.05	8.22	7.35	4.47	4.57	4.15	4.57	4.46	4.51	4.35
27	MAFIP	8177120	6.77	5.38	5.85	6.18	5.60	5.41	5.19	5.72	6.01	6.12	5.08	5.86	5.22	5.31	5.31	5.48
28	MXRA5P1	8176574	4.62	4.09	4.48	4.34	4.26	4.50	4.37	4.06	5.34	4.27	3.81	4.18	4.29	3.86	3.94	4.48
29	NLGN4Y	8176655	4.97	4.82	5.13	4.64	4.57	4.84	4.78	4.72	5.24	4.90	4.58	4.17	4.62	4.32	4.37	4.58
30	OFD1P5Y	8177195	3.71	3.68	3.87	3.52	3.67	3.36	3.51	3.45	4.13	3.63	3.61	3.35	3.91	3.69	3.66	3.67
		8176692	3.71	3.68	3.87	3.52	3.67	3.36	3.51	3.45	4.13	3.63	3.61	3.35	3.91	3.69	3.66	3.67

	Gene Name	Probesets	NI0627.CEL	VE9-0291.CEL	VE9-0336.CEL	VE9-0432.CEL	VE9-0472.CEL	VE9-0515.CEL	VE9-0567.CEL	VE9-0687.CEL	VE9-0817.CEL	VE9-1036.CEL	VE9-1050.CEL	VE9-0697.CEL	VE9-0739.CEL	VE9-0748.CEL	VE9-0307.CEL	VE9-0039.CEL
31	P2RY8	8177026	8.49	8.89	8.92	9.09	8.98	8.74	8.49	8.66	8.40	8.67	8.50	8.84	8.55	8.61	8.98	8.84
32	PCDH11Y	8176400	3.96	3.74	4.01	3.86	3.88	3.76	3.75	3.71	4.41	4.13	3.74	3.73	3.90	3.57	3.94	3.79
33	PCMTD2	8177460	6.59	7.25	6.61	7.58	7.05	7.56	7.50	7.66	5.25	6.75	8.26	7.27	7.67	7.73	7.16	7.23
		8176865	6.59	7.25	6.61	7.58	7.05	7.56	7.50	7.66	5.25	6.75	8.26	7.27	7.67	7.73	7.16	7.23
34	PLCXD1	8176286	6.29	6.69	6.79	6.82	7.04	6.49	6.74	6.88	6.98	6.30	6.49	6.05	6.57	6.89	6.72	6.10
35	PRKY	8176460	6.96	7.55	6.90	7.49	7.16	7.18	7.22	7.42	7.01	5.40	5.41	5.41	5.45	5.51	5.17	5.61
36	PRY	8176806	5.47	4.70	5.08	4.84	4.81	4.75	4.84	4.71	5.51	4.72	4.78	4.53	4.90	4.71	4.81	4.67
		8177323	5.45	4.67	5.05	4.84	4.78	4.75	4.81	4.74	5.51	4.76	4.76	4.50	4.87	4.74	4.84	4.66
		8176935	5.51	4.71	5.01	4.83	4.89	4.77	4.92	4.80	5.64	4.85	4.82	4.53	4.86	4.80	4.98	4.76
		8177395	5.51	4.71	5.01	4.83	4.89	4.77	4.92	4.80	5.64	4.85	4.82	4.53	4.86	4.80	4.98	4.76
37	RBMY1A3P	8177096	4.86	3.38	3.84	3.74	3.81	3.75	3.71	3.72	4.30	3.69	3.84	3.84	3.60	3.78	3.90	3.83
38	RBMY1B	8176742	4.29	2.94	3.19	3.06	3.08	3.16	2.98	2.87	3.69	2.98	3.00	2.98	3.17	3.24	3.07	3.11
		8177277	7.33	2.19	2.12	2.83	2.97	2.40	2.69	2.57	2.88	2.29	3.19	2.72	2.32	2.92	3.42	2.96
39	RBMY1F	8176753	4.50	3.03	3.36	3.16	3.18	3.15	3.17	2.99	3.75	3.07	3.15	3.01	3.20	3.31	3.22	3.27
		8177290	4.50	3.03	3.36	3.16	3.18	3.15	3.17	2.99	3.75	3.07	3.15	3.01	3.20	3.31	3.22	3.27
		8176766	4.50	3.05	3.35	3.16	3.19	3.17	3.15	3.01	3.76	3.05	3.12	3.05	3.24	3.32	3.25	3.22
		8176789	4.21	2.99	3.36	3.16	3.19	3.12	3.11	3.04	3.75	3.06	3.11	3.02	3.30	3.26	3.28	3.10
		8177331	4.45	3.02	3.36	3.18	3.19	3.19	3.15	3.01	3.81	3.08	3.11	3.05	3.27	3.34	3.28	3.20
40	RBMY2AP	8177389	3.80	3.25	3.20	3.18	3.22	3.13	3.42	3.32	3.81	3.49	3.28	3.26	3.13	3.50	3.32	3.38
		8176817	3.80	3.25	3.20	3.18	3.22	3.13	3.42	3.32	3.81	3.49	3.28	3.26	3.13	3.50	3.32	3.38
		8176943	3.80	3.25	3.20	3.18	3.22	3.13	3.42	3.32	3.81	3.49	3.28	3.26	3.13	3.50	3.32	3.38
		8176941	3.48	2.80	3.39	3.24	3.23	3.00	3.06	3.01	4.06	3.25	3.10	3.05	2.90	3.71	3.09	2.95
		8176815	3.48	2.80	3.39	3.24	3.23	3.00	3.06	3.01	4.06	3.25	3.10	3.05	2.90	3.71	3.09	2.95
		8177393	3.48	2.80	3.39	3.24	3.23	3.00	3.06	3.01	4.06	3.25	3.10	3.05	2.90	3.71	3.09	2.95
41	RBMY2NP	8177114	4.26	4.44	4.69	4.94	5.31	4.85	4.55	4.64	5.32	4.55	5.05	4.77	4.85	4.86	5.18	4.69
		8176427	4.26	4.44	4.69	4.94	5.31	4.85	4.55	4.64	5.32	4.55	5.05	4.77	4.85	4.86	5.18	4.69
42	RBMY2TP	8177280	3.44	3.29	3.87	3.89	3.65	3.65	3.79	3.59	4.40	4.44	4.17	3.86	3.61	3.61	4.03	4.12
43	RFTN1	8177072	4.52	4.44	4.49	4.27	4.35	4.26	4.21	4.08	4.56	4.25	4.16	4.40	4.38	4.52	4.41	4.20
44	RN5-8S6	8176568	5.00	5.40	4.88	5.30	5.33	5.05	5.24	5.31	5.11	5.16	5.50	5.00	5.39	5.39	4.84	5.54
45	RN5S518	8177116	4.91	4.97	5.59	4.86	4.80	4.72	4.64	4.72	5.64	5.86	5.00	4.80	4.81	4.93	5.04	5.19
46	RN5S519	8177118	5.82	5.31	5.88	5.28	5.28	5.48	4.92	4.77	6.26	5.81	5.41	5.35	5.61	4.65	5.37	5.25
47	RN5S520	8176667	6.46	5.91	6.16	6.12	6.10	5.83	5.94	5.83	6.91	6.15	6.45	5.54	6.41	6.00	6.05	6.11
		8177191	6.46	5.91	6.16	6.12	6.10	5.83	5.94	5.83	6.91	6.15	6.45	5.54	6.41	6.00	6.05	6.11
48	RN5S521	8177189	6.24	5.26	6.13	5.32	5.66	5.57	5.70	5.54	6.75	5.49	5.77	5.72	5.62	5.53	5.70	5.81
		8176669	6.24	5.26	6.13	5.32	5.66	5.57	5.70	5.54	6.75	5.49	5.77	5.72	5.62	5.53	5.70	5.81
49	RPS4Y1	8176375	8.62	8.65	8.20	8.51	8.98	8.79	8.20	8.75	7.10	4.19	3.94	3.91	3.55	3.58	3.90	3.78
50	RPS4Y2	8176730	5.05	5.04	4.87	5.20	5.39	4.91	5.29	5.53	4.88	4.38	3.79	3.64	4.09	4.03	4.08	3.96
51	SHOX	8176297	4.87	4.80	5.11	4.87	4.90	4.83	4.94	4.60	5.61	5.42	5.01	4.56	5.04	4.87	5.04	5.21
52	SLC25A6	8177003	9.76	10.00	10.08	10.03	10.27	10.06	9.77	9.96	9.37	9.44	9.35	9.63	9.69	9.61	10.13	9.77
53	SLC9B1	8177130	4.76	4.63	4.95	4.93	4.44	4.49	3.97	5.07	3.88	4.48	4.01	3.95	4.58	4.77	4.55	3.93
54	SPRY3	8176955	4.23	4.15	4.45	4.40	4.41	4.51	4.45	4.51	4.63	4.39	4.74	5.02	4.45	4.14	4.46	4.54
55	SRY	8177038	3.30	3.55	3.62	3.36	3.53	3.49	3.36	3.07	3.77	3.28	3.20	3.13	3.24	3.11	3.66	3.68
56	TBL1Y	8176442	5.06	4.83	4.89	4.74	4.91	4.74	4.81	4.67	5.07	4.75	4.85	4.78	4.99	4.61	4.84	4.99
57	TGIF2LY	8176397	3.77	3.77	4.06	3.75	3.53	3.68	3.67	3.66	4.22	4.43	3.79	3.92	3.71	3.69	3.94	3.67
58	TMSB4Y	8176644	5.82	6.05	6.12	5.70	5.78	6.16	5.80	5.63	6.17	5.52	5.63	5.45	5.76	5.55	5.48	5.51
59	TSPY1	8176494	6.04	4.82	4.90	4.96	4.86	4.76	4.92	4.73	5.24	4.88	4.84	4.77	4.84	4.97	4.85	4.72
		8176544	6.11	4.88	4.95	5.03	4.94	4.84	5.00	4.79	5.29	4.99	4.94	4.83	4.91	5.06	4.92	4.77
		8176508	6.11	4.85	4.92	4.99	4.91	4.81	4.97	4.77	5.27	4.95	4.91	4.80	4.88	5.03	4.90	4.76
60	TSPY14P	8176737	3.88	3.70	3.87	3.59	3.47	3.66	3.55	4.08	4.55	3.64	3.86	3.73	3.74	3.87	4.13	3.55

	Gene Name	Probesets	NI0627.CEL	VE9-0291.CEL	VE9-0336.CEL	VE9-0432.CEL	VE9-0472.CEL	VE9-0515.CEL	VE9-0567.CEL	VE9-0687.CEL	VE9-0817.CEL	VE9-1036.CEL	VE9-1050.CEL	VE9-0697.CEL	VE9-0739.CEL	VE9-0748.CEL	VE9-0307.CEL	VE9-0039.CEL
61	TSPY3	8176517	6.14	5.07	5.18	5.25	5.11	5.04	5.18	4.99	5.53	5.16	5.10	4.98	5.13	5.19	5.07	4.94
		8176524	6.11	4.88	4.96	5.06	4.95	4.84	5.01	4.81	5.29	5.01	4.95	4.82	4.92	5.04	4.91	4.80
62	TSPY4	8176484	6.11	4.87	4.91	4.98	4.90	4.81	4.97	4.79	5.29	4.98	4.88	4.81	4.86	5.01	4.92	4.78
		8176532	6.11	4.77	4.81	4.90	4.80	4.71	4.87	4.68	5.15	4.86	4.83	4.72	4.77	4.95	4.82	4.68
63	TTTY1	8177108	4.25	4.14	4.40	4.23	3.99	3.88	3.99	4.05	4.79	4.72	4.23	4.15	4.07	4.28	4.54	4.36
		8176429	4.25	4.14	4.40	4.23	3.99	3.88	3.99	4.05	4.79	4.72	4.23	4.15	4.07	4.28	4.54	4.36
64	TTTY10	8177261	5.15	5.33	5.52	5.30	5.20	5.65	5.48	5.31	5.89	4.23	3.78	4.00	4.40	4.16	4.21	4.03
65	TTTY11	8177085	3.39	3.55	3.59	3.59	3.55	3.53	3.56	3.59	3.87	3.32	3.48	3.46	3.58	3.58	3.67	3.44
66	TTTY12	8176471	4.71	3.86	4.10	3.40	4.08	3.72	3.79	3.56	4.45	3.49	3.58	3.74	4.01	3.77	4.03	3.64
67	TTTY13	8177282	5.22	4.52	4.76	4.62	4.81	4.38	4.57	4.40	5.57	4.58	4.88	4.83	4.79	4.82	4.54	4.63
68	TTTY14	8177214	3.76	3.89	3.89	3.33	3.47	3.70	3.54	3.66	3.43	3.43	3.14	3.24	3.32	3.20	3.43	3.21
		8177217	6.21	6.61	6.70	6.42	6.66	6.18	6.52	6.57	7.26	6.95	6.53	6.35	6.46	6.23	6.38	6.51
69	TTTY2	8177053	4.48	3.76	4.02	3.66	3.62	3.69	3.68	3.76	4.37	4.09	3.67	3.65	3.78	3.74	3.94	3.91
		8176558	4.48	3.76	4.02	3.66	3.62	3.69	3.68	3.76	4.37	4.09	3.67	3.65	3.78	3.74	3.94	3.91
70	TTTY5	8177344	5.37	5.01	5.25	4.72	5.08	4.92	4.98	4.79	5.57	5.07	5.14	4.92	5.26	4.98	5.37	5.14
71	TTTY6B	8176782	4.97	4.47	4.52	4.52	4.52	4.32	4.33	4.12	4.87	4.61	4.34	4.09	4.27	4.68	4.68	4.11
		8177347	4.97	4.47	4.52	4.52	4.52	4.32	4.33	4.12	4.87	4.61	4.34	4.09	4.27	4.68	4.68	4.11
72	TTTY7	8177101	4.91	4.69	4.65	4.28	4.28	4.50	4.58	4.19	5.33	4.79	4.43	4.27	4.53	4.87	5.10	4.84
		8176435	4.91	4.69	4.65	4.28	4.28	4.50	4.58	4.19	5.33	4.79	4.43	4.27	4.53	4.87	5.10	4.84
73	TTTY8	8177048	5.10	4.08	4.06	3.96	4.40	4.04	3.66	4.32	4.94	3.84	4.19	4.05	3.93	4.47	4.05	4.01
		8176553	5.10	4.08	4.06	3.96	4.40	4.04	3.66	4.32	4.94	3.84	4.19	4.05	3.93	4.47	4.05	4.01
74	TXLNG2P	8176698	7.03	7.87	7.41	7.69	7.41	7.47	7.51	7.55	6.52	4.59	4.11	4.39	4.33	4.12	4.08	4.24
		8176709	7.00	7.36	6.78	7.39	6.91	7.31	6.90	7.46	5.61	3.41	3.29	3.76	3.55	3.47	3.56	3.36
75	USP9Y	8176578	6.69	7.76	7.05	7.69	7.29	7.23	7.12	7.48	5.76	3.65	3.70	3.55	3.62	3.70	3.82	3.79
76	UTY	8177137	7.83	8.14	8.04	8.28	7.98	8.22	7.92	8.22	7.12	3.71	3.68	3.68	3.66	3.51	3.81	3.73
77	VAMP7	8176962	7.73	8.15	8.03	8.15	8.17	8.55	8.15	8.31	7.47	7.70	8.00	8.39	8.21	8.27	8.20	8.01
78	VCY	8176650	5.14	5.23	5.75	5.41	5.56	5.12	5.28	5.15	6.24	5.66	5.35	5.38	5.41	5.61	5.59	5.52
		8177169	5.14	5.23	5.75	5.41	5.56	5.12	5.28	5.15	6.24	5.66	5.35	5.38	5.41	5.61	5.59	5.52
79	XKRY	8177180	3.22	3.02	3.20	3.06	3.04	2.77	3.14	3.35	3.26	3.13	3.10	2.83	3.01	2.93	3.23	3.11
		8176675	3.22	3.02	3.20	3.06	3.04	2.77	3.14	3.35	3.26	3.13	3.10	2.83	3.01	2.93	3.23	3.11
80	ZBED1	8177029	7.71	7.78	7.87	7.72	7.80	7.87	7.87	7.87	7.87	7.70	7.73	7.88	7.79	7.76	7.86	7.73
81	ZFY	8176384	6.68	7.36	7.31	7.35	7.27	7.10	6.97	7.18	6.35	4.36	4.36	4.57	4.21	4.42	4.20	4.71
82	0	8176469	4.03	5.46	4.37	5.24	4.66	4.45	4.73	5.66	3.97	3.73	3.43	3.36	3.14	3.50	3.38	3.30
83	0	8177068	2.43	2.69	2.38	2.67	2.69	2.87	2.83	2.35	2.64	2.30	2.15	2.56	2.34	2.34	2.36	2.09
84	0	8176648	6.94	6.64	6.58	6.71	6.71	6.64	6.55	6.51	7.42	6.15	6.47	6.40	6.53	6.23	6.48	6.37
85	0	8177227	2.70	3.03	3.34	2.94	2.83	3.04	2.88	3.15	2.78	3.13	2.94	3.07	2.99	2.94	3.41	3.21
86	0	8177403	3.44	3.51	3.72	3.54	3.54	3.44	3.47	3.67	3.81	4.00	3.64	3.62	3.64	3.64	4.11	3.51
87	0	8176931	3.44	3.51	3.72	3.54	3.54	3.44	3.47	3.67	3.81	4.00	3.64	3.62	3.64	3.64	4.11	3.51
88	0	8177092	4.82	4.53	4.51	4.22	4.47	4.07	4.67	4.37	5.31	4.23	4.36	3.89	4.10	4.57	4.72	4.30
89	0	8176476	2.36	2.71	3.07	2.49	2.61	2.53	2.44	2.53	2.98	2.81	2.67	3.05	2.74	2.71	3.00	2.80
90	0	8176663	2.99	3.04	3.14	2.97	3.26	2.71	2.87	2.97	3.02	3.20	3.01	2.98	3.13	2.94	3.52	3.32

	Gene Name	Probesets	NI0627.CEL	VE9-0291.CEL	VE9-0336.CEL	VE9-0432.CEL	VE9-0472.CEL	VE9-0515.CEL	VE9-0567.CEL	VE9-0687.CEL	VE9-0817.CEL	VE9-1036.CEL	VE9-1050.CEL	VE9-0697.CEL	VE9-0739.CEL	VE9-0748.CEL	VE9-0307.CEL	VE9-0039.CEL
91	0	8177176	2.99	3.04	3.14	2.97	3.26	2.71	2.87	2.97	3.02	3.20	3.01	2.98	3.13	2.94	3.52	3.32
92	0	8176779	4.17	4.26	4.56	4.31	4.16	4.43	4.47	4.20	4.61	4.79	4.49	4.42	4.27	4.44	4.62	4.43
93	0	8177044	3.53	3.88	4.16	3.68	3.61	3.84	3.74	3.74	4.06	4.44	3.81	4.21	3.60	4.04	3.77	3.94
94	0	8176419	6.25	5.17	5.28	5.36	5.24	5.16	5.31	5.09	5.67	5.32	5.21	5.14	5.23	5.42	5.23	5.10
95	0	8176679	3.95	4.27	4.16	3.95	3.75	4.27	3.80	4.09	4.29	4.02	4.09	3.82	3.89	3.92	4.01	3.99
96	0	8177212	3.95	4.27	4.16	3.95	3.75	4.27	3.80	4.09	4.29	4.02	4.09	3.82	3.89	3.92	4.01	3.99
97	0	8176415	2.25	3.10	2.99	2.96	2.56	2.79	2.84	2.31	2.91	2.97	2.73	2.80	3.02	2.72	2.86	2.98
98	0	8176677	5.99	5.54	5.82	5.79	5.67	5.65	6.02	5.43	6.31	5.92	6.06	5.63	5.74	5.62	5.27	5.81
99	0	8177178	5.99	5.54	5.82	5.79	5.67	5.65	6.02	5.43	6.31	5.92	6.06	5.63	5.74	5.62	5.27	5.81
100	0	8176576	3.84	3.76	3.95	3.58	3.56	3.58	3.82	3.55	4.65	3.60	4.20	3.52	3.73	3.60	3.60	3.91
101	0	8177303	4.23	3.13	3.34	3.21	3.25	3.16	3.30	3.11	3.71	3.24	3.25	3.14	3.18	3.42	3.30	3.34
102	0	8176478	2.96	2.87	3.23	2.99	2.78	3.15	3.07	3.03	3.32	2.89	2.85	3.23	2.89	2.93	3.05	3.20
103	0	8176949	4.39	4.83	5.39	4.64	4.72	4.58	4.44	4.47	4.98	5.85	4.72	5.01	4.62	4.06	4.95	5.20
104	0	8177387	3.39	2.53	2.89	2.12	2.52	2.44	2.48	2.80	2.91	2.33	2.25	3.29	2.43	2.72	2.68	2.20
105	0	8176947	3.39	2.53	2.89	2.12	2.52	2.44	2.48	2.80	2.91	2.33	2.25	3.29	2.43	2.72	2.68	2.20
106	0	8177083	4.80	3.61	3.74	3.92	3.82	3.76	3.53	4.02	4.24	4.49	3.79	3.93	3.98	4.37	3.70	3.94
107	0	8177174	2.02	2.22	2.51	1.98	2.05	2.18	2.23	2.06	2.15	2.11	2.05	2.43	2.21	2.28	2.28	2.13
108	0	8177184	2.47	2.83	2.76	2.69	2.82	2.58	3.02	2.81	3.03	2.70	2.44	2.92	2.49	3.18	2.74	2.55
109	0	8176673	2.47	2.83	2.76	2.69	2.82	2.58	3.02	2.81	3.03	2.70	2.44	2.92	2.49	3.18	2.74	2.55
110	0	8177193	5.21	3.88	4.45	3.85	3.90	4.09	3.91	3.85	4.90	4.70	4.10	4.21	4.23	4.33	4.42	4.29
111	0	8176665	5.21	3.88	4.45	3.85	3.90	4.09	3.91	3.85	4.90	4.70	4.10	4.21	4.23	4.33	4.42	4.29
112	0	8176480	2.54	2.54	2.79	2.62	2.61	2.75	2.72	2.61	2.84	2.83	2.46	2.68	2.56	2.78	2.80	2.53
113	0	8176566	9.79	9.58	10.12	9.76	9.71	9.40	9.95	9.48	9.78	10.37	10.05	9.68	9.76	9.39	9.47	10.06
114	0	8177070	2.47	2.68	2.62	2.93	2.65	2.50	2.66	2.76	2.79	2.67	2.54	2.77	2.43	2.90	2.71	2.39
115	0	8176986	5.53	5.87	6.11	5.53	5.88	5.54	5.82	5.72	6.42	6.20	5.81	5.69	5.51	5.80	6.02	5.91
116	0	8176373	3.65	4.00	3.79	3.66	3.62	3.81	3.83	3.89	4.05	3.99	3.63	3.95	3.80	3.77	4.32	3.83
117	0	8176395	10.61	11.12	10.60	11.32	11.18	10.51	11.32	10.46	10.80	11.21	11.36	10.70	10.96	10.75	10.32	10.94
118	0	8177401	3.40	3.51	3.38	3.25	3.60	3.47	3.34	3.56	4.09	3.26	3.57	3.62	3.52	3.70	3.43	3.76
119	0	8176933	3.40	3.51	3.38	3.25	3.60	3.47	3.34	3.56	4.09	3.26	3.57	3.62	3.52	3.70	3.43	3.76
120	0	8176482	5.27	5.18	5.20	4.93	5.40	5.00	5.19	4.83	5.05	5.19	5.56	4.81	5.02	5.45	5.11	4.97
121	0	8176921	8.84	8.28	8.32	7.86	8.13	7.83	8.42	8.08	8.88	8.62	8.50	8.05	8.25	8.23	8.17	8.41
122	0	8177424	8.84	8.28	8.32	7.86	8.13	7.83	8.42	8.08	8.88	8.62	8.50	8.05	8.25	8.23	8.17	8.41
123	0	8177079	2.61	2.49	2.96	3.15	2.51	2.68	2.53	2.43	2.40	2.71	2.53	2.62	2.79	2.52	2.85	2.59
124	0	8177081	4.63	4.06	4.37	4.10	3.90	4.09	4.16	3.92	4.65	4.42	4.30	4.06	3.98	4.18	4.24	4.39
