# Supplementary material for: A novel approach for human whole transcriptome analysis based on absolute gene expression of microarray data
Source: PeerJ. 2017 Dec 8;5:e4133. doi: 10.7717/peerj.4133 (PMC5724404; doi:10.7717/peerj.4133)
Supplement: Table S3 — Fluorescence intensities obtained for DDX3Y, EIF1AY and TXLNG2P genes in male (M) and female (F) subjects using the Affymetrix HuGene 1.0 ST microarray (GSE89571). [file peerj-05-4133-s003.pdf]

| | Sex | DDX3Y | EIF1AY | TXLNG2P | |
|---|---|---|---|---|---|
| **ID/Probesets** | | **8176624** | **8176719** | **8176709** | **8176698** |
| NI0627.CEL | M | 8.81 | 7.65 | 7.00 | 7.03 |
| VE9-0291.CEL | M | 9.19 | 8.51 | 7.36 | 7.87 |
| VE9-0336.CEL | M | 9.10 | 8.39 | 6.78 | 7.41 |
| VE9-0432.CEL | M | 9.44 | 8.63 | 7.39 | 7.69 |
| VE9-0472.CEL | M | 9.27 | 8.40 | 6.91 | 7.41 |
| VE9-0515.CEL | M | 9.51 | 9.19 | 7.31 | 7.47 |
| VE9-0567.CEL | M | 9.16 | 8.79 | 6.90 | 7.51 |
| VE9-0687.CEL | M | 9.46 | 9.14 | 7.46 | 7.55 |
| VE9-0817.CEL | M | 8.09 | 7.86 | 5.61 | 6.52 |
| VE9-0039.CEL | F | 4.45 | 2.93 | 3.36 | 4.24 |
| VE9-0307.CEL | F | 3.99 | 3.25 | 3.56 | 4.08 |
| VE9-0697.CEL | F | 4.24 | 3.46 | 3.76 | 4.39 |
| VE9-0739.CEL | F | 4.15 | 3.37 | 3.55 | 4.33 |
| VE9-0748.CEL | F | 4.30 | 3.11 | 3.47 | 4.12 |
| VE9-1036.CEL | F | 4.21 | 2.83 | 3.41 | 4.59 |
| VE9-1050.CEL | F | 4.13 | 3.14 | 3.29 | 4.11 |
