# Supplementary material for: A novel approach for human whole transcriptome analysis based on absolute gene expression of microarray data
Source: PeerJ. 2017 Dec 8;5:e4133. doi: 10.7717/peerj.4133 (PMC5724404; doi:10.7717/peerj.4133)
Supplement: Table S4 — The fluorescence intensities of the genes were obtained by the HuGene 1.0 ST microarray of Affymetrix (GSE89571) for male (M) and female (F) samples. [file peerj-05-4133-s004.pdf]

| | sex | COX6A1 | COX6A1 | CYC1 | GAPDH | GSK3A | H6PD | LTBP4 | MDH1 | MTX1 | SDHA | VEGFB | SRY |
|---|---|---|---|---|---|---|---|---|---|---|---|---|---|
| **ID/Probesets** | | **7959153** | **8119153** | **8148728** | **7953385** | **8037152** | **7897441** | **8028872** | **8042259** | **7905968** | **8104166** | **7940904** | **8177038** |
| NI0627.CEL | M | 7.91 | 7.98 | 7.18 | 11.74 | 7.08 | 5.91 | 6.62 | 7.18 | 6.09 | 9.17 | 7.05 | 3.30 |
| VE9-0291.CEL | M | 8.02 | 8.00 | 7.27 | 11.00 | 7.93 | 6.72 | 6.89 | 7.03 | 6.06 | 9.50 | 7.56 | 3.55 |
| VE9-0336.CEL | M | 7.71 | 7.70 | 7.24 | 11.34 | 7.56 | 6.33 | 6.94 | 6.68 | 6.06 | 9.20 | 7.50 | 3.62 |
| VE9-0432.CEL | M | 8.01 | 8.05 | 7.40 | 11.11 | 7.69 | 6.34 | 6.81 | 7.12 | 5.86 | 9.24 | 7.36 | 3.36 |
| VE9-0472.CEL | M | 8.13 | 8.15 | 7.69 | 11.50 | 7.87 | 6.37 | 6.72 | 7.29 | 5.98 | 9.30 | 7.52 | 3.53 |
| VE9-0515.CEL | M | 8.22 | 8.14 | 7.53 | 11.80 | 7.52 | 6.22 | 6.44 | 7.40 | 6.22 | 9.36 | 7.23 | 3.49 |
| VE9-0567.CEL | M | 8.09 | 8.05 | 7.36 | 11.05 | 7.96 | 6.44 | 6.79 | 6.78 | 6.07 | 9.12 | 7.28 | 3.36 |
| VE9-0687.CEL | M | 8.22 | 8.23 | 7.41 | 11.44 | 7.71 | 6.48 | 6.65 | 7.28 | 6.04 | 9.33 | 7.29 | 3.07 |
| VE9-0817.CEL | M | 7.78 | 7.89 | 6.89 | 11.17 | 7.37 | 6.40 | 7.47 | 5.87 | 6.38 | 8.44 | 7.47 | 3.77 |
| VE9-1036.CEL | F | 7.81 | 7.85 | 6.94 | 11.07 | 7.69 | 6.39 | 6.96 | 6.64 | 6.07 | 8.85 | 7.36 | 3.28 |
| VE9-1050.CEL | F | 8.02 | 8.01 | 7.35 | 11.16 | 7.74 | 6.38 | 6.78 | 6.82 | 5.85 | 8.97 | 7.04 | 3.20 |
| VE9-0697.CEL | F | 7.91 | 7.88 | 6.96 | 11.13 | 7.54 | 6.43 | 6.49 | 6.94 | 5.96 | 9.42 | 7.13 | 3.13 |
| VE9-0739.CEL | F | 8.06 | 8.02 | 7.27 | 11.45 | 7.50 | 6.54 | 6.94 | 6.92 | 6.24 | 8.95 | 7.19 | 3.24 |
| VE9-0748.CEL | F | 7.71 | 7.75 | 6.94 | 11.07 | 7.78 | 6.48 | 6.52 | 6.51 | 6.22 | 8.91 | 7.20 | 3.11 |
| VE9-0307.CEL | F | 8.16 | 8.12 | 7.21 | 11.68 | 7.81 | 6.46 | 6.63 | 6.95 | 6.01 | 9.10 | 7.62 | 3.66 |
| VE9-0039.CEL | F | 7.96 | 7.96 | 7.15 | 11.04 | 7.76 | 6.28 | 6.71 | 6.71 | 6.23 | 9.06 | 7.39 | 3.68 |
