# Supplementary material for: A novel approach for human whole transcriptome analysis based on absolute gene expression of microarray data
Source: PeerJ. 2017 Dec 8;5:e4133. doi: 10.7717/peerj.4133 (PMC5724404; doi:10.7717/peerj.4133)
Supplement: Table S5 [file peerj-05-4133-s005.pdf]

ID	Sex	TXNIP	B2M	PYHIN1	ZZZ3	BEND7	CAPN11	ZNF99	ABCA6	UBE2U	C3orf30
NI0627.CEL	M	12.61	12.30	7.32	6.89	4.40	4.79	3.48	3.32	3.16	3.07
VE9-0291.CEL	M	12.85	12.18	7.86	7.70	4.07	4.50	3.57	3.58	2.91	3.11
VE9-0336.CEL	M	12.79	11.94	7.21	7.37	4.35	4.54	3.66	3.69	3.13	3.25
VE9-0432.CEL	M	12.84	12.23	7.83	7.75	4.06	4.26	3.44	3.55	2.79	3.24
VE9-0472.CEL	M	12.75	12.16	7.57	7.47	4.32	4.15	3.39	3.47	2.80	3.01
VE9-0515.CEL	M	13.01	12.41	7.84	7.46	4.42	4.02	3.50	3.63	2.92	3.00
VE9-0567.CEL	M	12.93	12.41	7.09	7.28	4.60	4.16	3.45	3.39	3.02	3.03
VE9-0687.CEL	M	12.90	12.37	7.91	7.55	4.66	4.29	3.59	3.58	2.83	3.14
VE9-0817.CEL	M	12.69	11.77	6.51	6.25	4.99	4.96	3.59	3.81	3.13	3.23
VE9-1036.CEL	F	12.53	11.96	6.58	7.28	4.28	4.26	3.44	3.40	2.85	3.07
VE9-1050.CEL	F	12.54	12.37	6.87	6.99	4.92	4.48	3.40	3.58	2.91	3.06
VE9-0697.CEL	F	12.91	12.40	7.76	7.67	4.41	4.39	3.56	3.35	3.02	3.15
VE9-0739.CEL	F	12.77	12.35	7.02	7.21	4.52	4.29	3.27	3.33	2.91	3.07
VE9-0748.CEL	F	12.85	12.05	7.37	7.39	4.21	4.35	3.46	3.59	2.98	3.21
VE9-0307.CEL	F	12.69	12.08	7.44	7.28	4.54	4.50	3.80	3.61	3.04	3.51
VE9-0039.CEL	F	12.81	12.17	6.77	7.77	4.29	4.43	3.41	3.71	2.98	3.26
