# Supplementary material for: A novel approach for human whole transcriptome analysis based on absolute gene expression of microarray data
Source: PeerJ. 2017 Dec 8;5:e4133. doi: 10.7717/peerj.4133 (PMC5724404; doi:10.7717/peerj.4133)
Supplement: Table S6 — Gene expression data were normalized according to the reference gene (Glyceraldehyde 3-phosphate dehydrogenase (GAPDH)). The ID samples are listed in the first column. Cp, crossing point; RelExp, relative expression. [file peerj-05-4133-s006.pdf]

ID	Sex	qPCR	DDX3Y	EIF1AY	TXLNG2P
NI0627	M	Ct	27.51	28.34	25.57
		Rel Exp	2.48E-02	1.39E-02	9.53E-02
VE9-0291	M	Ct	26.74	26.04	24.99
		Rel Exp	2.51E-02	4.08E-02	8.46E-02
VE9-0336	M	Ct	25.67	24.93	24.49
		Rel Exp	3.35E-02	5.61E-02	7.62E-02
VE9-0432	M	Ct	25.97	25.64	24.76
		Rel Exp	5.14E-02	6.48E-02	0.1191
VE9-0472	M	Ct	26.15	26.48	24.34
		Rel Exp	3.88E-02	3.09E-02	0.1362
VE9-0515	M	Ct	25.63	26.30	24.11
		Rel Exp	2.99E-02	1.89E-02	8.59E-02
VE9-0567	M	Ct	26.44	25.13	25.55
		Rel Exp	2.57E-02	6.39E-02	4.76E-02
VE9-0687	M	Ct	25.84	26.26	24.75
		Rel Exp	3.19E-02	2.40E-02	6.81E-02
VE9-0817	M	Ct	26.51	25.73	25.50
		Rel Exp	3.33E-02	5.71E-02	6.66E-02
VE9-0039	F	Ct	32.52	34.17	38.35
		Rel Exp	7.30E-04	2.33E-04	1.29E-05
VE9-0307	F	Ct	31.42	34.09	40.00
		Rel Exp	1.39E-03	2.18E-04	3.63E-06
VE9-0697	F	Ct	31.73	33.58	38.82
		Rel Exp	1.57E-03	4.36E-04	1.16E-05
VE9-0739	F	Ct	30.22	32.70	37.13
		Rel Exp	1.52E-03	2.73E-04	1.26E-05
VE9-0748	F	Ct	31.94	33.69	36.79
		Rel Exp	9.34E-04	2.77E-04	3.23E-05
VE9-1036	F	Ct	32.78	33.54	35.81
		Rel Exp	4.90E-04	2.88E-04	5.98E-05
VE9-1050	F	Ct	31.95	33.15	35.70
		Rel Exp	5.55E-04	2.42E-04	4.13E-05
