# Supplementary material for: A novel approach for human whole transcriptome analysis based on absolute gene expression of microarray data
Source: PeerJ. 2017 Dec 8;5:e4133. doi: 10.7717/peerj.4133 (PMC5724404; doi:10.7717/peerj.4133)
Supplement: Table S7 — Gene expression data were normalized according to the reference gene (Glyceraldehyde 3-phosphate dehydrogenase (GAPDH)). The ID samples are listed in the first column. Cp, crossing point; RelExp, relative expression. [file peerj-05-4133-s007.pdf]

ID	Sex	qPCR	TXNIP	B2M	ZZZ3	PYHIN1	CAPN11	BEND7	ZNF299	ABCA6	C3ORF30	UBE2U
NI0627	M	Ct	21.56	18.72	27.55	25.60	32.51	26.77	36.27	33.60	40.00	35.33
		Rel Exp	1.528	11.01	2.41E-02	9.30E-02	7.73E-04	4.15E-02	5.74E-05	3.65E-04	4.31E-06	1.10E-04
VE9-0291	M	Ct	20.54	17.33	26.46	24.48	33.56	40.00	34.47	33.11	0.00	40.00
		Rel Exp	1.844	17.07	3.05E-02	0.1202	2.22E-04	2.56E-06	1.18E-04	3.03E-04	0	2.56E-06
VE9-0336	M	Ct	19.76	16.73	25.69	23.78	34.55	37.35	40.00	32.48	40.00	0.00
		Rel Exp	2.02	16.49	3.31E-02	0.1245	7.11E-05	1.03E-05	1.63E-06	3.00E-04	1.63E-06	0
VE9-0432	M	Ct	19.95	16.86	26.04	23.85	34.89	34.50	40.00	32.10	0.00	0.00
		Rel Exp	3.332	28.56	4.91E-02	0.2245	1.07E-04	1.39E-04	3.08E-06	7.35E-04	0	0
VE9-0472	M	Ct	19.94	17.66	25.79	24.10	34.70	0.00	36.16	31.85	0.00	0.00
		Rel Exp	2.881	13.92	5.00E-02	0.1604	1.04E-04	0	3.77E-05	7.47E-04	0	0
VE9-0515	M	Ct	18.67	16.99	25.20	23.42	32.98	34.29	37.13	31.24	0.00	0.00
		Rel Exp	3.726	11.97	4.02E-02	0.1389	1.83E-04	7.42E-05	1.03E-05	6.15E-04	0	0
VE9-0567	M	Ct	19.90	16.78	26.60	24.58	35.69	39.46	35.79	32.56	0.00	40.00
		Rel Exp	2.392	20.83	2.30E-02	9.33E-02	4.22E-05	3.10E-06	3.95E-05	3.70E-04	0	2.13E-06
VE9-0687	M	Ct	19.77	17.16	25.82	24.03	32.46	33.73	36.80	31.34	0.00	0.00
		Rel Exp	2.143	13.14	3.24E-02	0.1124	3.26E-04	1.35E-04	1.61E-05	7.08E-04	0	0
VE9-0817	M	Ct	19.80	16.64	26.66	24.31	33.94	33.76	36.04	32.25	40.00	40.00
		Rel Exp	3.477	31.08	3.00E-02	0.1522	1.92E-04	2.18E-04	4.50E-05	6.23E-04	2.89E-06	2.89E-06
VE9-0039	F	Ct	20.14	17.93	27.09	25.96	34.65	37.21	35.80	33.17	0.00	0.00
		Rel Exp	3.918	18.11	3.16E-02	6.93E-02	1.68E-04	2.84E-05	7.56E-05	4.66E-04	0	0
VE9-0307	F	Ct	21.30	18.15	27.56	25.47	33.66	37.37	40.00	33.23	0.00	0.00
		Rel Exp	1.55	13.75	2.01E-02	8.61E-02	2.93E-04	2.25E-05	3.63E-06	3.95E-04	0	0
VE9-0697	F	Ct	20.61	17.07	27.09	24.89	33.31	34.78	40.00	36.10	0.00	0.00
		Rel Exp	3.512	40.63	3.92E-02	0.1802	5.25E-04	1.90E-04	5.10E-06	7.64E-05	0	0
VE9-0739	F	Ct	19.82	16.66	26.25	24.44	33.16	33.70	40.00	32.02	40.00	40.00
		Rel Exp	2.048	18.34	2.39E-02	8.35E-02	1.98E-04	1.37E-04	1.73E-06	4.36E-04	1.73E-06	1.73E-06
VE9-0748	F	Ct	20.92	17.80	26.99	25.48	35.02	34.12	36.69	32.77	0.00	0.00
		Rel Exp	1.931	16.85	2.88E-02	8.18E-02	1.10E-04	2.05E-04	3.46E-05	5.24E-04	0	0
VE9-1036	F	Ct	21.67	17.79	27.47	26.04	34.68	31.43	40.00	36.16	40.00	40.00
		Rel Exp	1.079	15.9	1.94E-02	5.24E-02	1.31E-04	1.25E-03	3.28E-06	4.71E-05	3.28E-06	3.28E-06
VE9-1050	F	Ct	21.03	17.01	27.60	0.00	34.67	34.87	35.91	32.63	36.65	0.00
		Rel Exp	1.072	17.39	1.13E-02	0	8.40E-05	7.35E-05	3.57E-05	3.47E-04	2.13E-05	0
