# Supplementary material for: A novel approach for human whole transcriptome analysis based on absolute gene expression of microarray data
Source: PeerJ. 2017 Dec 8;5:e4133. doi: 10.7717/peerj.4133 (PMC5724404; doi:10.7717/peerj.4133)
Supplement: Table S8 [file peerj-05-4133-s008.pdf]

Sample #	ID
sample1	NI0627.CEL
sample2	VE9-0039.CEL
sample3	VE9-0291.CEL
sample4	VE9-0307.CEL
sample5	VE9-0336.CEL
sample6	VE9-0432.CEL
sample7	VE9-0472.CEL
sample8	VE9-0515.CEL
sample9	VE9-0567.CEL
sample10	VE9-0687.CEL
sample11	VE9-0697.CEL
sample12	VE9-0739.CEL
sample13	VE9-0748.CEL
sample14	VE9-0817.CEL
sample15	VE9-1036.CEL
sample16	VE9-1050.CEL
