# Supplementary material for: A novel approach for human whole transcriptome analysis based on absolute gene expression of microarray data
Source: PeerJ. 2017 Dec 8;5:e4133. doi: 10.7717/peerj.4133 (PMC5724404; doi:10.7717/peerj.4133)
Supplement: Table S9 — Complete list of significant enriched KEGG pathways (p ≤ E–9) obtained after analyzing the total 5% most expressed leukocyte genes of 16 subjects. [file peerj-05-4133-s009.pdf]

Ranking	Pathway Name	# Gene	p value
1	Ribosome	61	7.62E-64
2	Phagosome	52	5.67E-35
3	Regulation of actin cytoskeleton	54	3.43E-29
4	Antigen processing and presentation	31	9.22E-24
5	Fc gamma R-mediated phagocytosis	33	6.49E-23
6	Leishmaniasis	29	3.37E-22
7	Chemokine signaling pathway	43	1.62E-21
8	Osteoclast differentiation	36	1.92E-21
9	Protein processing in endoplasmic reticulum	40	3.21E-21
10	Natural killer cell mediated cytotoxicity	35	1.5E-19
11	Endocytosis	41	7.79E-19
12	Pathways in cancer	52	8.53E-19
13	Leukocyte transendothelial migration	30	5.74E-17
14	Focal adhesion	38	1.76E-16
15	Hematopoietic cell lineage	26	2.24E-16
16	Viral myocarditis	23	1.05E-15
17	Shigellosis	21	7.19E-15
18	Pathogenic Escherichia coli infection	20	1.45E-14
19	Toxoplasmosis	29	1.57E-14
20	B cell receptor signaling pathway	22	5.77E-14
21	Staphylococcus aureus infection	19	1.3E-13
22	T cell receptor signaling pathway	25	3.26E-13
23	Cell adhesion molecules (CAMs)	27	9.31E-13
24	Rheumatoid arthritis	22	3.84E-12
25	Allograft rejection	15	4.08E-12
26	Jak-STAT signaling pathway	28	6.1E-12
27	MAPK signaling pathway	37	7.93E-12
28	Spliceosome	25	1.22E-11
29	Neurotrophin signaling pathway	25	1.22E-11
30	Long-term potentiation	19	1.25E-11
31	Graft-versus-host disease	15	1.95E-11
32	Type I diabetes mellitus	15	4.23E-11
33	Prostate cancer	20	1.33E-10
34	Chagas disease (American trypanosomiasis)	21	3.59E-10
35	Autoimmune thyroid disease	15	8.5E-10
36	Fc epsilon RI signaling pathway	18	9.4E-10
37	Renal cell carcinoma	17	9.55E-10
38	Lysosome	22	9.55E-10
39	Bacterial invasion of epithelial cells	17	9.55E-10
40	Systemic lupus erythematosus	23	1.64E-09
41	Chronic myeloid leukemia	17	1.82E-09
42	Insulin signaling pathway	23	2.11E-09
43	Vascular smooth muscle contraction	21	2.4E-09
44	Epithelial cell signaling in Helicobacter pylori	16	4.68E-09

	infection		
45	Hepatitis C	22	6.23E-09
