# Supplementary material for: A novel approach for human whole transcriptome analysis based on absolute gene expression of microarray data
Source: PeerJ. 2017 Dec 8;5:e4133. doi: 10.7717/peerj.4133 (PMC5724404; doi:10.7717/peerj.4133)
Supplement: Table S10 — Complete list of significant enriched KEGG pathways (p ≤ E–9) obtained after analyzing the total 5% most expressed genes liver cells arrays in GSE18269. [file peerj-05-4133-s010.pdf]

Ranking	Pathway Name	# Gene	p value
1	Metabolic pathways	266	2.57E-148
2	Ribosome	76	4.39E-95
3	Oxidative phosphorylation	60	4.07E-51
4	Parkinson's disease	59	2.5E-50
5	Huntington's disease	64	4.26E-46
6	Protein processing in endoplasmic reticulum	60	1.97E-44
7	Alzheimer's disease	58	1.1E-41
8	Complement and coagulation cascades	38	9.19E-37
9	Drug metabolism - cytochrome P450	30	3.5E-24
10	Fatty acid metabolism	23	6.2E-22
11	Valine, leucine and isoleucine degradation	23	1.15E-21
12	Metabolism of xenobiotics by cytochrome P450	27	8.03E-21
13	Glycolysis / Gluconeogenesis	25	1.8E-19
14	Protein export	16	4.54E-18
15	Arginine and proline metabolism	22	7.13E-18
16	Peroxisome	25	3.6E-17
17	Glycine, serine and threonine metabolism	17	2.19E-16
18	Retinol metabolism	22	4.47E-16
19	PPAR signaling pathway	22	3.75E-15
20	Propanoate metabolism	16	5.9E-15
21	Cardiac muscle contraction	22	3.23E-14
22	RNA transport	28	9.25E-13
23	Tryptophan metabolism	16	1.04E-12
24	Citrate cycle (TCA cycle)	14	1.13E-12
25	Phagosome	28	1.15E-12
26	Proteasome	16	2.17E-12
27	Pyruvate metabolism	15	6.83E-12
28	Tyrosine metabolism	15	1.01E-11
29	Pathogenic Escherichia coli infection	17	1.05E-11
30	Glutathione metabolism	16	1.84E-11
31	beta-Alanine metabolism	11	1.44E-10
32	Butanoate metabolism	12	4.36E-10
33	Drug metabolism - other enzymes	15	4.36E-10
34	Steroid hormone biosynthesis	15	1.34E-09
35	Primary bile acid biosynthesis	9	2.21E-09
36	Bile secretion	16	5.23E-09
37	Spliceosome	21	5.81E-09
