# Supplementary figures and images for: A novel approach for human whole transcriptome analysis based on absolute gene expression of microarray data
Source: PeerJ. 2017 Dec 8;5:e4133. doi: 10.7717/peerj.4133 (PMC5724404; doi:10.7717/peerj.4133)

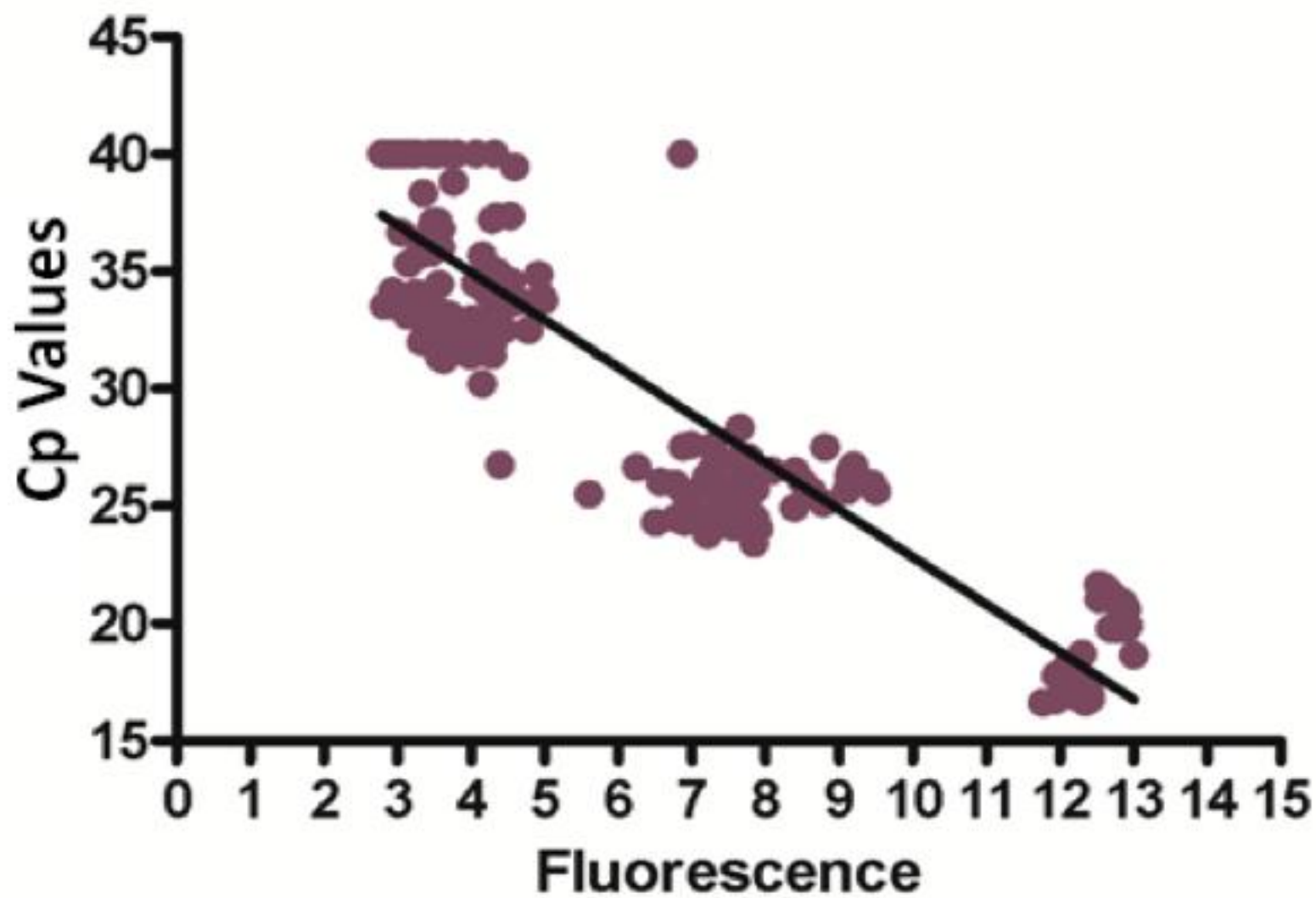

Supplement: Figure S1 — The expression values of 13 different genes are showed in the graph. The expression values by microarrays (fluorescence) are represented in the X axis, and the expression values by RT-qPCR (Cp Values) are represented on the Y axis. The Spearman correlation was r = − 0.882, p = 2.84–69. [file peerj-05-4133-s011.pdf]

**Frequency**

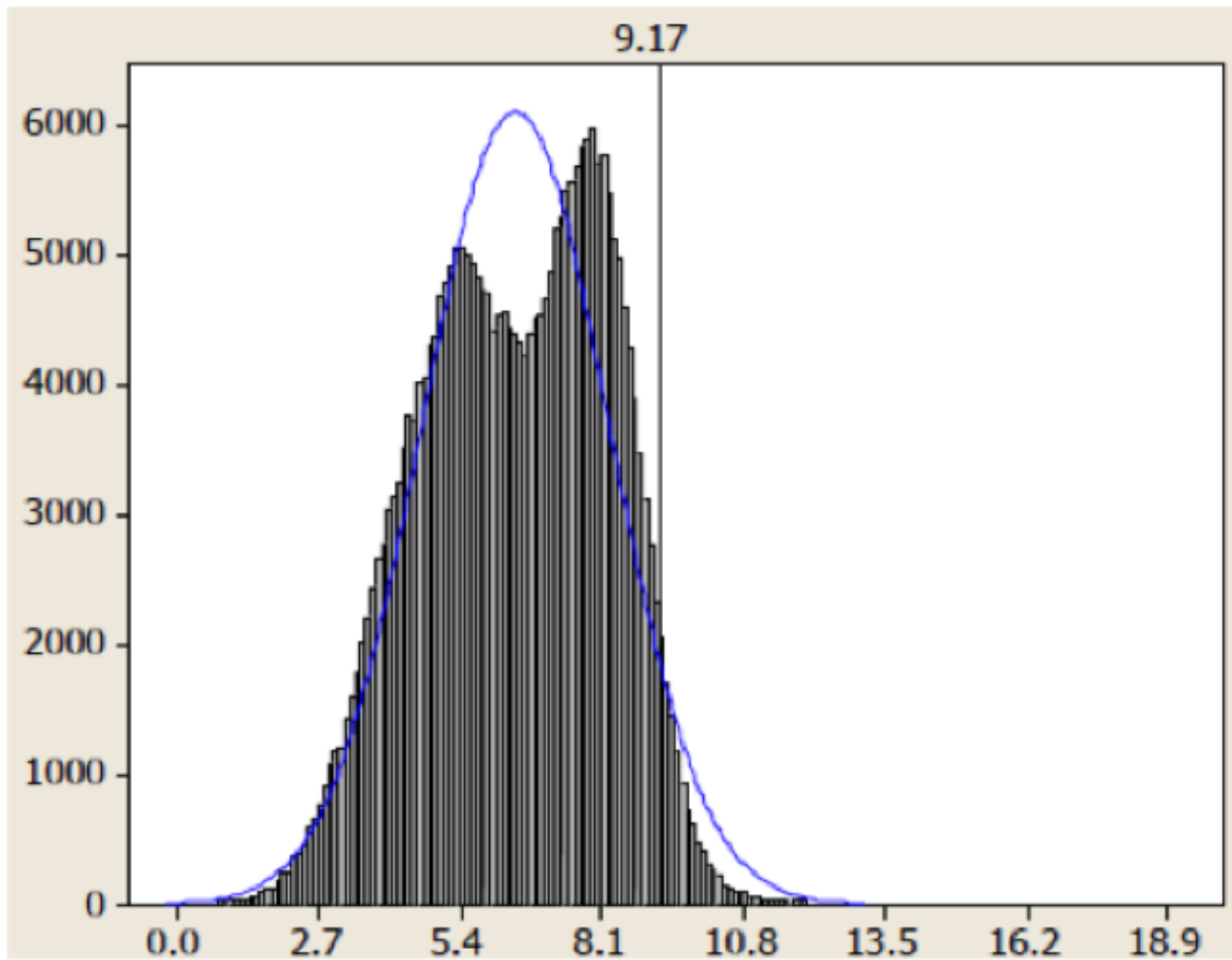

**Log2 Fluorescence**

Supplement: Figure S2 — The distribution of the fluorescence intensities are showed and the fluorescence value for the absolute gene expression threshold is marked by the vertical line in graph (absolute gene expression threshold = 9.17). All probes with fluorescence under this value were considered as not-expressed (97.9% (n = 53,466)). The raw data of fluorescence was converted in log2 to obtain a clearer histogram. [file peerj-05-4133-s012.pdf]
