# Supplementary material for: A novel approach for human whole transcriptome analysis based on absolute gene expression of microarray data
Source: PeerJ. 2017 Dec 8;5:e4133. doi: 10.7717/peerj.4133 (PMC5724404; doi:10.7717/peerj.4133)
Supplement: Figure S3 — In the left circle, we showed the number (558) and percent (1%) of probes that are not expressed by Barcode (Shyamsundar et al., 2005; Lipshutz et al., 1999; Tang et al., 2007) and are different to the non-expressed probes by the absolute gene expression threshold. In the second circle and on the right side is showing the number (2828) and the percent (5%) of probes that are not expressed by the absolute gene expression threshold and are different to the non-expressed probes by Barcode (Shyamsundar et al., 2005; Lipshutz et al., 1999; Tang et al., 2007). In the middle of both circles are the number (50638) and percent (93.7%) of the probes that are non-expressed by both Barcode and the absolute gene expression threshold. [file peerj-05-4133-s013.pdf]

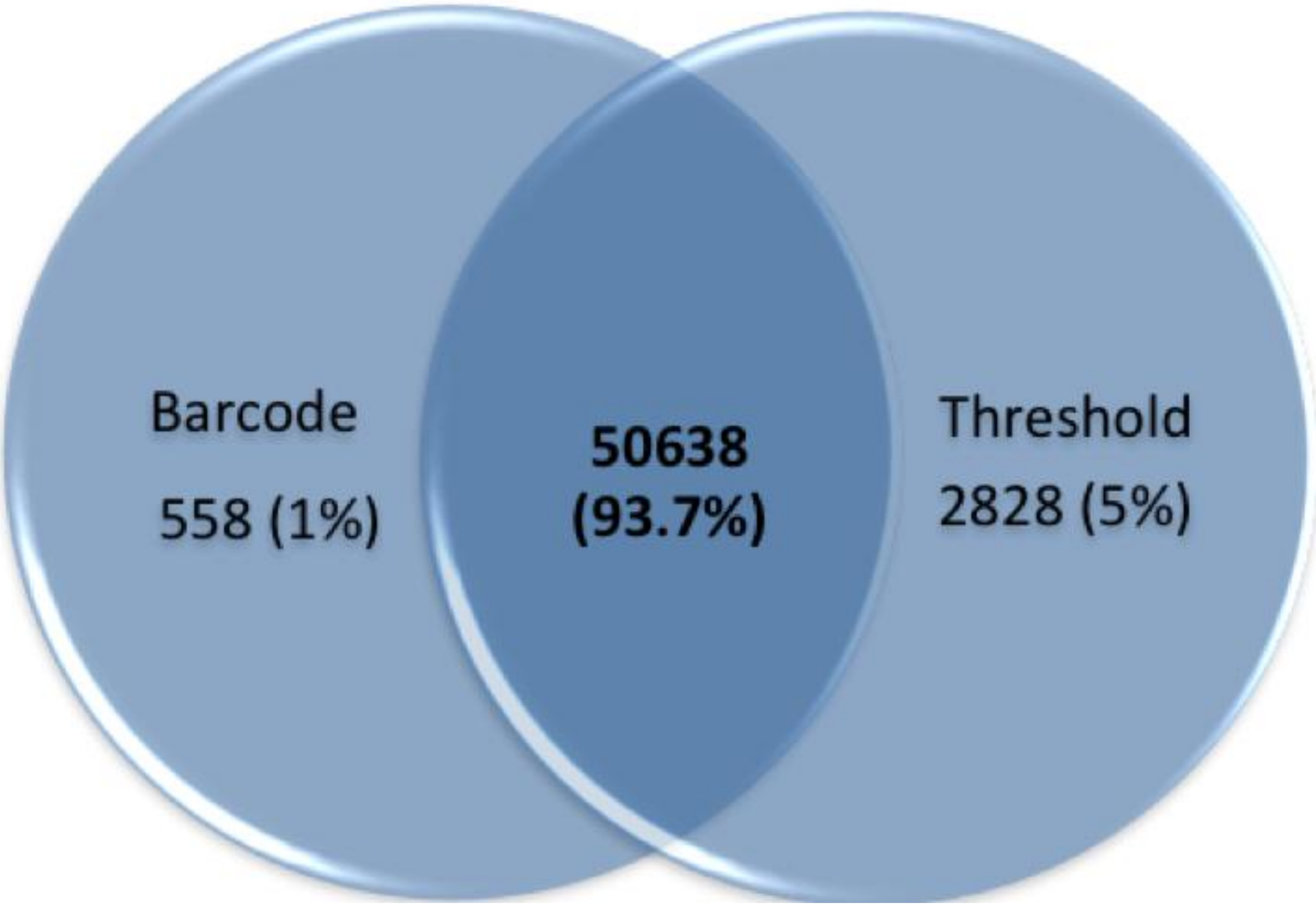

Barcode
558 (1%)
50638
(93.7%)
Threshold
2828 (5%)
