# Supplementary material for: A novel approach for human whole transcriptome analysis based on absolute gene expression of microarray data
Source: PeerJ. 2017 Dec 8;5:e4133. doi: 10.7717/peerj.4133 (PMC5724404; doi:10.7717/peerj.4133)
Supplement: Supplemental Information 1 [file peerj-05-4133-s014.pdf]

Miame Checklist

Manuscript: “A novel approach for human whole transcriptome profiling based on absolute gene expression analysis of microarray data”

Shirley Bikel, Leonor Jacobo-Albavera, Fausto Sánchez-Muñoz, Fernanda Cornejo-Granados, Samuel Canizales-Quinteros, Xavier Soberón, Rogerio R. Sotelo-Mundo, Blanca E del Río-Navarro, Alfredo Mendoza-Vargas, Filiberto Sánchez, and Adrián Ochoa-Leyva.

Part 1 Experiment description

Type of experiment:
Absolute expression analysis of leukocytes from human samples at a single time point.
Experimental variables:
None.
n-count:
16 samples (9 males and 7 female subjects).
tissues used for slide:
Human blood tissue.
other variables (wean weight, pooled samples, etc.)
None.

Part 2 Array design.

Array series: Affymetrix Gene Chip Human Gene 1.0 ST Array.
Deconvoluted spot list with gene names: https://www.thermofisher.com/order/catalog/product/901085
Array type (mouse, human, cDNA, oligo, number of genes): human; cDNA; 21,014 genes
Array size: 5μm.
Slide type (and coating): The array consists of a square glass substrate mounted in a plastic cartridge. The glass contains an array of oligonucleotides that, when mounted, is on the inner glass surface. A chamber in the plastic housing directly under the glass acts as a reservoir where hybridization and washing occur.

Part 3 Samples

Cy3/Cy5 labels for tissues: None.
Dye swap? Or reference control?: None.
Labelling protocol used: Standard Affymetrix protocol. Performed at Affymetrix platform in the Genotyping and Expression Analysis Unit of the National Institute of Genomic Medicine (INMEGEN).
Sample extraction protocol used: RNA was isolated using TRIzol (Invitrogen) according to manufacturer’s protocol.
Amount of sample labeled: Standard Affymetrix protocol. Performed at Affymetrix platform in the Genotyping and Expression Analysis Unit of the National Institute of Genomic Medicine (INMEGEN).

Part 4 Hybridizations

Hybridization protocol: Standard Affymetrix protocol. Performed at Affymetrix platform in the Genotyping and Expression Analysis Unit of the National Institute of Genomic Medicine (INMEGEN).
ALL modifications and deviations from the protocol: None.
Manual hybridization or automatic chamber: Automatic chamber.
Number of slides done at the same time: 16.

Hyb time: 16 hours.
Number of washes: Standard Affymetrix protocol. Performed at Affymetrix platform in the Genotyping and Expression Analysis Unit of the National Institute of Genomic Medicine (INMEGEN).
Amount of labelled sample hybridized: Standard Affymetrix protocol. Performed at Affymetrix platform in the Genotyping and Expression Analysis Unit of the National Institute of Genomic Medicine (INMEGEN).
Labelling efficiency: Acceptable according to the standard Affymetrix protocol. Performed at Affymetrix platform in the Genotyping and Expression Analysis Unit of the National Institute of Genomic Medicine (INMEGEN).

Part 5 Measurements

Which version of scanner software used: Affymetrix GeneChip Command Console Software (AGCC).
Laser power for scan: Default parameters of the Affymetrix Gene Chip Scanner 3000 7G.
Instrument model numbers: Model number 00-0210**,** Affymetrix Gene Chip Scanner 3000 7G.
Must save original .tiff format images (composite image is optional): These data are available on the NCBI Gene Expression Omnibus (GEO) under accession number GSE89571.
Normalization protocol: Robust Multi-Array (RMA).
Does the scanner software subtract background? How much?. Default parameters of the Affymetrix GeneChip Command Console Software (AGCC).
Spot raw values, background intensity, ch1 and 2 intensity, etc. Default parameters of the Affymetrix GeneChip Command Console Software (AGCC).
Corresponding gene name. Default parameters of the Affymetrix GeneChip Command Console Software (AGCC).
Methods of analysis (MAN, Spotfire, Genespring) be detailed. Default parameters of the Affymetrix GeneChip Command Console Software (AGCC).
Normalized to controls? Controls removed? All normalization parameters. Normalization was made using the Robust Multi-Array (RMA) under default parameters in RMA Express software.
Name of Images, Experiment, and location of files. These data are available on the NCBI Gene Expression Omnibus (GEO) under accession number GSE89571.
Lowess or other normalization if used (and parameters). Normalization was made using the Robust Multi-Array (RMA) under default parameters in RMA Express software.
Output file: None.
Normalized ratios: None.
Numerical manipulations: None.
Cut off values: None.

Part 6 Normalization controls

Hypothesis: The normalized fluorescence values were used to develop an absolute gene expression method for microarray analysis.
Gene expression patterns found: None.
Controls used, normalization methods used (see above): Normalization was made using the Robust Multi-Array (RMA) under default parameters in RMA Express software.
